# MicroRNA-29a promotes the proliferation of human nasal epithelial cells and inhibits their apoptosis and promotes the development of allergic rhinitis by down-regulating FOS expression

**DOI:** 10.1371/journal.pone.0255480

**Published:** 2021-08-12

**Authors:** Yuqin Fan, Zhiyuan Tang, Jie Sun, Xiaorui Zhao, Zhen Li, Yiqing Zheng, Xianhai Zeng, Juan Feng

**Affiliations:** 1 Department of Otolaryngology-Head and Neck Surgery, Shanghai Key Laboratory of Translational Medicine on Ear and Nose Diseases, Shanghai Ninth People’s Hospital, Shanghai Jiao Tong University School of Medicine, Ear Institute, Shanghai Jiao Tong University School of Medicine, Shanghai, China; 2 Department of Otorhinolaryngology, Shenzhen Longgang E.N.T Hospital & Shenzhen Key Laboratory of E.N.T., Institute of E.N.T., Shenzhen, Guangdong, China; 3 Department of Otorhinolaryngology, The Eighth Affiliated Hospital of Sun Yat-sen University, Shenzhen, Guangdong, China; 4 Department of Otorhinolaryngology, The First Affiliated Hospital of Xinjiang Medical University, Urumqi, Xinjiang, China; 5 Department of Otolaryngology, Sun Yat-Sen Memorial Hospital, Guangzhou, Guangdong, P.R. China; University of Ulsan College of Medicine, REPUBLIC OF KOREA

## Abstract

**Objective:**

To explore the regulation of microRNA-29a (miR-29a) on FOS in human nasal epithelial cells and its molecular mechanism, as well as the effects of miR-29a on the cell proliferation and apoptosis.

**Methods:**

By cell transfection, gene silencing, quantitative real-time polymerase chain reaction (qRT-PCR), flow cytometry and TUNEL assay (for cell apoptosis), CCK-8 assay (for cell proliferation), dual-luciferase reporter gene assay and Western Blot, it was validated that miR-29a promoted the proliferation of human nasal epithelial cells and inhibited their apoptosis by down-regulating FOS expression in RPMI2650 and HNEpC cell lines.

**Results:**

①Compared with healthy controls, miR-29a expression was up-regulated and FOS mRNA expression was down-regulated in the nasal tissues from the patients with allergic rhinitis (AR). ②MiR-29a over-expression promoted the proliferation of RPMI2650 cells and HNEpC cells but inhibited their apoptosis. ③MiR-29a targeted at FOS. ④MiR-29a over-expression and FOS silencing both significantly promoted cell proliferation and inhibited cell apoptosis. After transfection with both miR-29a and FOS, there was a decrease in the proliferation but an increase in the apoptosis of cells.⑤MiR-29a promoted the proliferation of human nasal epithelial cells and inhibited their apoptosis by down-regulating FOS expression.

**Conclusion:**

MiR-29a-/FOS axis can be regarded as a potential marker and a new therapy for AR.

## Introduction

Allergic rhinitis (AR) is a kind of type I respiratory allergic disease caused by allergens, mediated by IgE and involving several types of immunocompetent cells and cytokines.

AR has such typical symptoms as sneezing, rhinorrhea, rhinocleisis and rhinocnesmus, which seriously affects the working, learning, and living quality of patients [1]. There are many therapeutic drugs for AR, including antihistaminics, corticosteroids, decongestants and nasal anticholinergic drugs; however, these drugs have serious side effects or poor efficacy [2]. Therefore, it is a key to find out new targets for the diagnosis and treatment of AR. As reported, gene therapy has a certain potential in treating allergic airway diseases including AR [3]. Besides, nasal cells with abnormal apoptosis are also an important target to treat AR [4].

In the preliminary study, we investigated the gene expression profile in the nasal tissues from AR and non-AR patients and screened out totally 230 differentially expressed genes (97 genes with expression up-regulation and 133 genes with expression down-regulation), of which FOS expression was down-regulated in AR [5]. FOS is a nuclear phosphoprotein produced by c-fos transcription and plays an important role in regulating cell proliferation, differentiation and apoptosis [6]. By analyzing TargetScan online database, we found that there was a microRNA-29a (miR-29a) binding site at 3’ untranslational region (UTR) of FOS gene (http://www.targetscan.org/).

MicroRNA (miRNA), a short RNA molecule consisting of 19–25 nucleotides, can regulate the post-transcriptional silencing of target genes. Single miRNAs can target at several hundreds of mRNAs, and influence the expression of multiple genes usually involved in the functional interaction pathways. The current studies have proven that miRNA is associated with the pathogenesis of many allergic diseases including asthma, eosinophilic esophagitis, AR and eczema [7–11].

However, the regulatory mechanisms of miRNA and mRNA in AR have not been completely clarified. This study aimed to explore the regulatory mechanism of miR-29a in inducing the proliferation and apoptosis of RPMI2650 (ATCC® CCL-30™) human nasal epithelial cell line via FOS.

## Materials and methods

### Clinical specimens

Patients presenting with AR visited Department of Otorhinolaryngology, Shenzhen Longgang E.N.T. hospital Guangdong, China from 2017 to 2019. The study population comprised 54 patients with clinical data on persistent AR and 30 controls. A detailed evaluation was conducted, including clinical history and physical examination, in patients and healthy controls. The diagnosis of AR was based on the patients medical history, symptoms, and the presence of a positive skin prick test (SPT, Allergopharma, Hamburg, Germany) in response to a panel of common allergens defined by the ARIA 2008 guidelines.1 The SPT results were utilized to diagnose AR in accordance with the recommendations of the Subcommittee on Allergen Standardization and Skin Tests of the European Academy of Allergy and Clinical Immunology. A positive SPT result was defined as the formation of a wheal larger than or equal to a half the diameter of the histamine control wheal, and at least 3 mm larger than the diameter of the negative control wheal. A total of 18 inhaled allergens were tested, including house dust, grass, tree, mold, food, and cat/dog dander. Exclusion criteria were: bronchial asthma, chronic rhinosinusitis, nasal polyposis, excessive septal deviation, and current smoking. The patients were not under pharmacological treatment (anti-histamines) at least 10 days before testing. Exclusion criteria for the healthy subjects were clinical history of rhinitis and positive reaction to any of the allergens from the test panel. All the patients and controls underwent a bronchial provocation test. Airway responsiveness to methacholine was evaluated, with FEV1 higher than 70% of predicted.

### Cell culture

RPMI2650 (ATCC® CCL-30™) and HNEpC (BNCC340481) human nasal epithelial cell lines were selected. The cells were statically cultured with RPMI 1640 (Gibco, Rockville, MD) culture medium containing 10% fetal bovine serum (FBS) (Sigma-Aldrich, St. Louis, MO), 100 U/mL penicillin and 100 mg/mL streptomycin in a cell incubator (Thermo Fisher Scientific, Waltham) of 37°C, 5% CO_2_ and a saturated humidity. The cells in the logarithmic phase were chosen for experiment.

### Transfection

At 1 day before transfection, the passage culture of HNEpC cells and RPMI 2650 cells was completed, and then the cells were cultured in a 6-well plate for lentivirus transfection [1]. Lentivirus particles were fabricated by Shanghai GeneChem Biotech Co., Ltd. According to different treatment methods, HNEpC cells and RPMI 2650 cells were separately divided into 5 groups: (1) control group: the cells were normally cultured, without treatment; (2) miR-29a over-expression negative control group (NC1 group): the cells were transfected with miR-29a over-expression negative control vector; (3) miR-29a over-expression group (miR-29a group): the cells were transfected with miR-29a over-expression vector; (4) miR-29a silencing negative control group (NC2 group): the cells were transfected with miR-29a inhibitor negative control; (5) miR-29a silencing group (si-miR-29a group): the cells were transfected with miR-29a inhibitor. The lentivirus sequences were as follows: NC1 (5’-UUCUCCGAACGUGUCACGUTT-3’), miR-29a (5’-UAGCACCAUCUGAAAU CGGUUA-3’), NC2 (5’-CAGUACUUUUGUGUAGUACAA-3’) and si-miR-29a (5’-UAACCGAUUUCAGAUGGUGCUA-3’).

### Detection of miR-29a transfection efficiency by real-time polymerase chain reaction (RT-PCR)

The total RNA (OD260/OD280 = 1.8–2.0 indicated that RNA purity was qualified) was extracted from cells using TRIzol kit (Takara, Dalian, China). The reverse transcription of mRNA and miRNA was performed with PrimeScript^TM^ RT Master Mix (TaKaRa, Japan) and miScript Reverse Transcription Kit (Qiagen, Germany), respectively. Quantitative RT-PCR (qRT-PCR) was conducted using Mastercycler® nexus X2 (Eppendorf, Hamburg, Germany) with the reaction conditions of 95°C 15s, 60°C 60s and 72°C 40s (35 cycles). The data were processed with 2^-ΔΔCt^ method, and the relative expression levels of miR-29a and FOS mRNA were calculated with GAPDH as the internal reference. The sequences of various primers are shown in Table 1.

**Table 1:** Primer sequences.

Name	Sequence
miR-29a	Forward:5′-ACACTCCAGCTGGGTAGCACCATCT GAAATC -3′
	Reverse: 5′- CTCAACTGGTGTCGTGGA -3′
FOS	Forward: 5′- CTACTACCATTCCCCAGCCG -3′
	Reverse: 5′- CTACTACCATTCCCCAGCCG -3′
GAPDH	Forward: 5′- TGCACCACCAACTGCTTAG‐-3′
	Reverse: 5′- GATGCAGGGATGATGTTC -3′

### Detection of cell proliferation by CCK8 assay

The cells in the logarithmic phase were inoculated into a 96-well plate by a density of 2×10^4^/ml and 100 μl/well, and then cultured in a 37°C and 5% CO_2_ incubator. At 24h, 48h and 72h, 10 μl CCK-8 solution (DOJINDO, Japan) was added into each well and mixed evenly. After 4h continuous culture, the zeroing was finished using the blank control wells, and the optical density (OD) of various wells was measured at 450 nm using a microplate reader.

### Detection of cell apoptosis by flow cytometry

After treatment, the cells were cultured for 24h, collected, re-suspended once using pre-cooled phosphate buffer solution (PBS) (4°C), centrifuged at 1,000 r/min for 5-10min, and washed. Thereafter, they were added with 300μl binding buffer for suspension, then added with 5μl Annexin V-APC and mixed evenly, and finally incubated at room temperature and protecting from light for 15 min. 5μl propidium iodide (PI) was added for staining at 5min before injection and 200μl binding buffer was added immediately before injection. The samples were eventually detected by flow cytometry (Beckman Coulter, Brea, CA), and the results were analyzed using CellQuest software (BD Bioscience, San Diego, CA).

### Detection of cell apoptosis by TUNEL assay

The cell slides were prepared with a 6-well plate. The cells were treated according to the experimental conditions in each group, washed with PBS for twice, added with 4% paraformaldehyde and then fixed at room temperature for 30min, and washed with distilled water for 2min × 2 times. Subsequently, they were membranolysed for 2min using 0.5ml 0.1% Triton X-100 prepared with 0.1mol/L citrate sodium buffer solution (pH 6.0), washed with PBS for 2min × 2 times, and added with 0.1ml pre-prepared TUNEL reaction mixture. Then, they were incubated in a 37°C and 5% CO_2_ incubator and protecting from light for 1h, washed for 2min × 2 times, incubated with DAPI (4’,6-diamidino-2-phenylindole dihydrochloride) and protecting from light for 5min, washed with PBS for 3 times, and photographed under fluorescence microscope.

### Detection of FOS, Bcl-2, Bax, Beclin1 and p62 protein expression levels in the cells by Western Blot

The nasal tissues in AR group and NR group were prepared into a homogenate and centrifuged at 2,000 r/min for 20min, and the supernatant was collected. The protein concentration was measured using BCA kit (Solarbio, Beijing, China), 40μl protein sample and 10% SDS gel buffer were mixed by 1:1 and heated 5min at 95°C for protein denaturation. Then, the mixture was transferred onto a polyvinylidene difluoride (PVDF) membrane at 80V (Merck, Darmstadt, Germany) for 30 min, and then the PVDF membrane was blocked with TBST solution containing 5% defatted milk powder at 4°C for 1h, added with rabbit anti-human FOS (1:1000, ab190289, Abcam), Bcl-2 (1:1000, ab196495, Abcam), Bax (1:1000, ab263897, Abcam), Beclin1 (1:1000, ab62557, Abcam), p62 (1:1000, ab155686, Abcam), and β-actin (1:2000, orb178392, Biorbyt, Cambridge, UK) polyclonal antibodies which were diluted with TBST solution containing 3% FBS protein, and then incubated overnight at 4°C. After re-warming, the PVDF membrane was incubated with horseradish peroxidase (HRP) labeled goat anti-rabbit IgG (1:1000, ABIN101988, antibodies-online, Aachen, Germany) for 1h, washed, and developed with ECL luminescent substrate for 3-5min. The protein expression level was normalized using β-actin, and the gray scan and quantification were performed with Image J(NIH) software.

### Dual-luciferase reporter gene assay

Wild-type (WT) and mutant-type (Mut) 3’UTRs of miR-29a and FOS were separately amplified from pGL3/luciferase vector (Promega, Madison, WI) and cloned into the downstream of luciferase reporter gene. After 48h transfection, luciferase activity in 293T cells (Shanghai Institute of Cell Biology, Chinese Academy of Sciences) was detected with the luciferase reporter gene system (Promega) according to the requirements in the instruction [2].

### RT-PCR

To further validate the effects of miR-29a on the growth and apoptosis of HNEpC cells and RPMI 2650 cells by targeting at FOS, the cells were divided into the following groups: (1) control group; (2) miR-29a over-expression group (miR-29a group); (3) FOS silencing negative control group (NC3 group); (4) FOS silencing group (si-FOS group); (5) miR-29a over-expression + FOS over-expression negative control group (miR-29a+NC4 group); (6) miR-29a over-expression + FOS over-expression group (miR-29a+FOS group). FOS transfection efficiency was detected by qRT-PCR, and the above experiment was repeated.

### Statistical analysis

SPSS 22.0 statistical analysis software was used for data processing, and the data analysis results were presented as mean ± standard deviation (SD). The data analysis between two groups was performed with *t* test and that among multiple groups was conducted using one-way analysis of variance (ANOVA). The subsequent analysis was implemented with Dunnett-t test, and the correlation in the expression between miR-29a and KLF4 was analyzed using Pearson correlation analysis. *P*<0.05 suggested that a difference was statistically significant.

The human studies informed consent of all participating subjects was obtained conform to internationally accepted standards.The consent obtained from study participants was verbal and have been approved by the ethical committee of Shenzhen Longgang District Hospital of Otolaryngology (Shenzhen Institute of Otolaryngology).

## Results

MiR-29a expression was up-regulated but FOS mRNA expression was down-regulated in the nasal tissues from AR patients, as compared with healthy controls.

**Fig 1:**
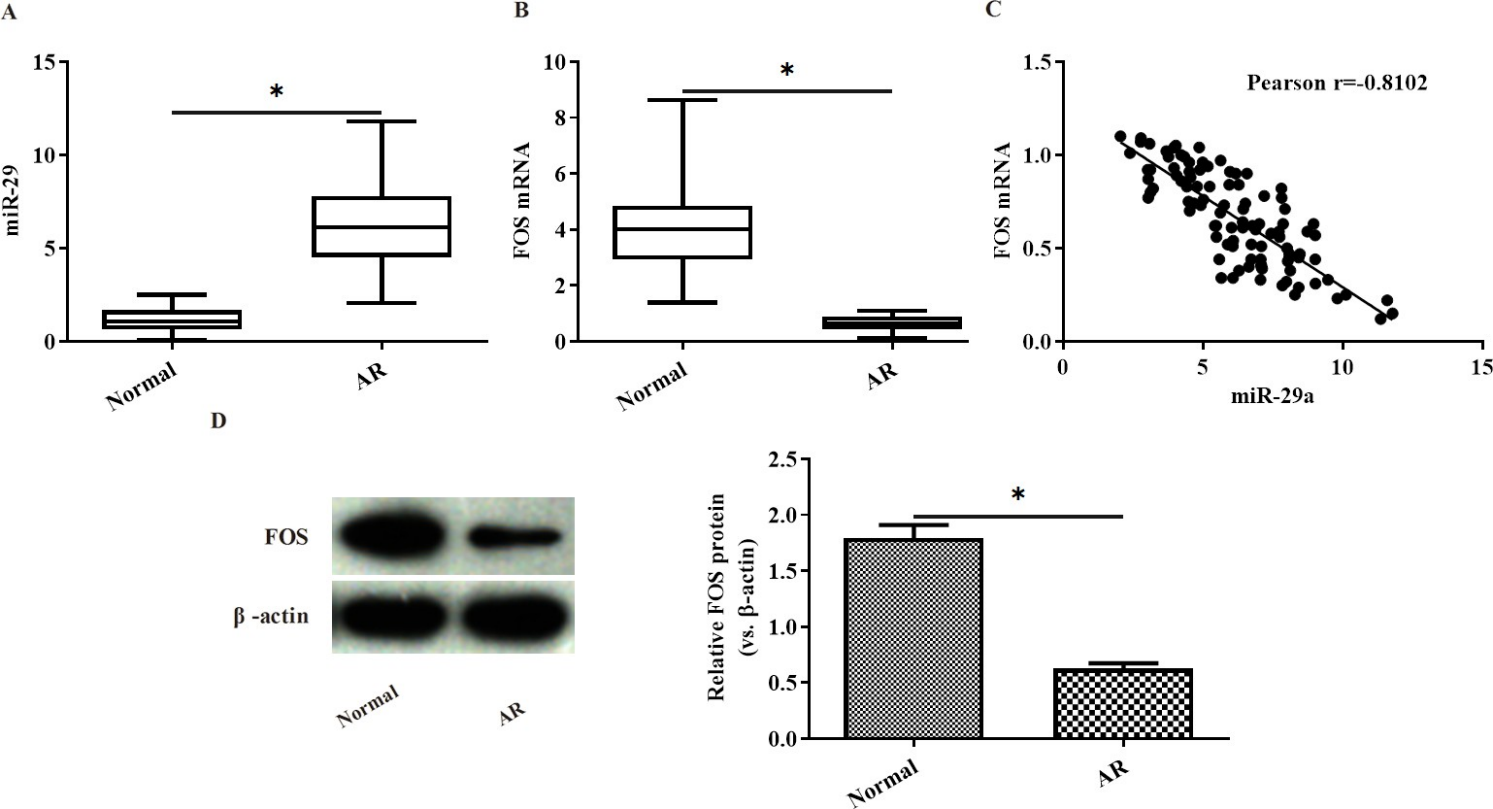
The expression of miR-29a and FOS mRNA in the nasal tissues from AR patients and healthy controls. (A-B) The expression of miR-29a and FOS mRNA in the nasal tissues from AR patients and healthy controls was analyzed by PCR; (C) the correlation in the expression between miR-29a and FOS mRNA was analyzed by Pearson correlation analysis; (D) FOS protein expression in the nasal tissues from AR patients and healthy controls was analyzed by western Blot. *p<0.05.

Compared with healthy controls, the level of miR-29a and FOS mRNA in the nasal tissues from AR patients was significantly increased and decreased, respectively (p<0.05) (Fig 1A and 1B). As shown by Pearson correlation analysis, there was a negative correlation in the expression between miR-29a and FOS mRNA in the nasal tissues from AR patients (r = -0.8102) (Fig 1C). Consistent with PCR results, FOS protein expression level in the nasal tissues from AR patients was significantly lower than that from normal controls (p<0.05) (Fig 1D).

MiR-29a over-expression promoted the proliferation of RPMI2650 cells and HNEpC cells but inhibited their apoptosis.

**Fig 2:**
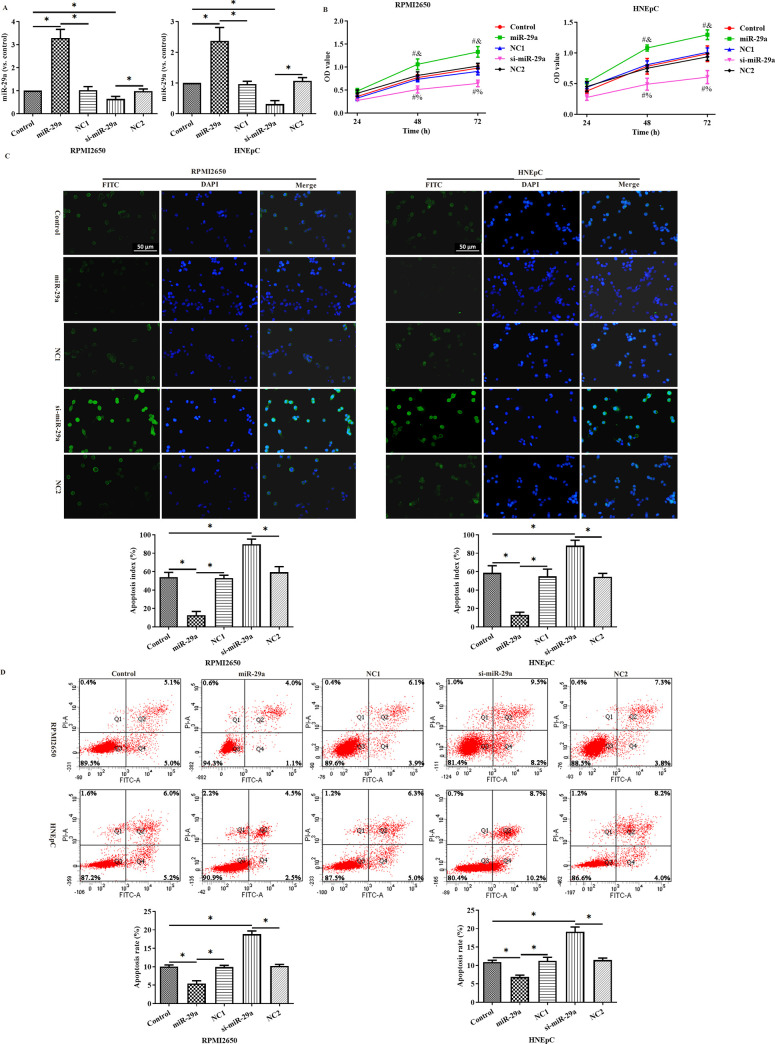
Mir-29a over-expression promoted the proliferation of RPMI2650 cells and HNEpC cells but inhibited their apoptosis. (A) MiR-29a transfection efficiency was validated by PCR; (B) the effects of miR-29a on the proliferation of RPMI2650 cells and HNEpC cells were analyzed by CCK8 assay; (C-D) the effects of miR-29a on the apoptosis of RPMI2650 cells and HNEpC cells were validated by flow cytometry and TUNEL assay. Compared with control group, #p<0.05; compared with NC1 group, &p<0.05; compared with NC2 group, %p<0.05.*:p<0.05.

After transfection with miR-29a over-expression and silencing vectors, we analyzed the transfection efficiency by PCR (Fig 2A). The level of miR-29a in miR-29a group was significantly higher than that in control group and NC1 group. Compared with control group and NC2 group, miR-29a level was markedly decreased in si-miR-29a group. The above findings indicated that miR-29a over-expression and silencing vectors were successfully transfected into RPMI2650 cells and HNEpC cells. The results of CCK8 assay showed that miR-29a over-expression could remarkably increase the OD of the above cells and promoted their proliferation (Fig 2B), but significantly inhibited their apoptosis (Fig 2C and 2D).

**Fig 3:**
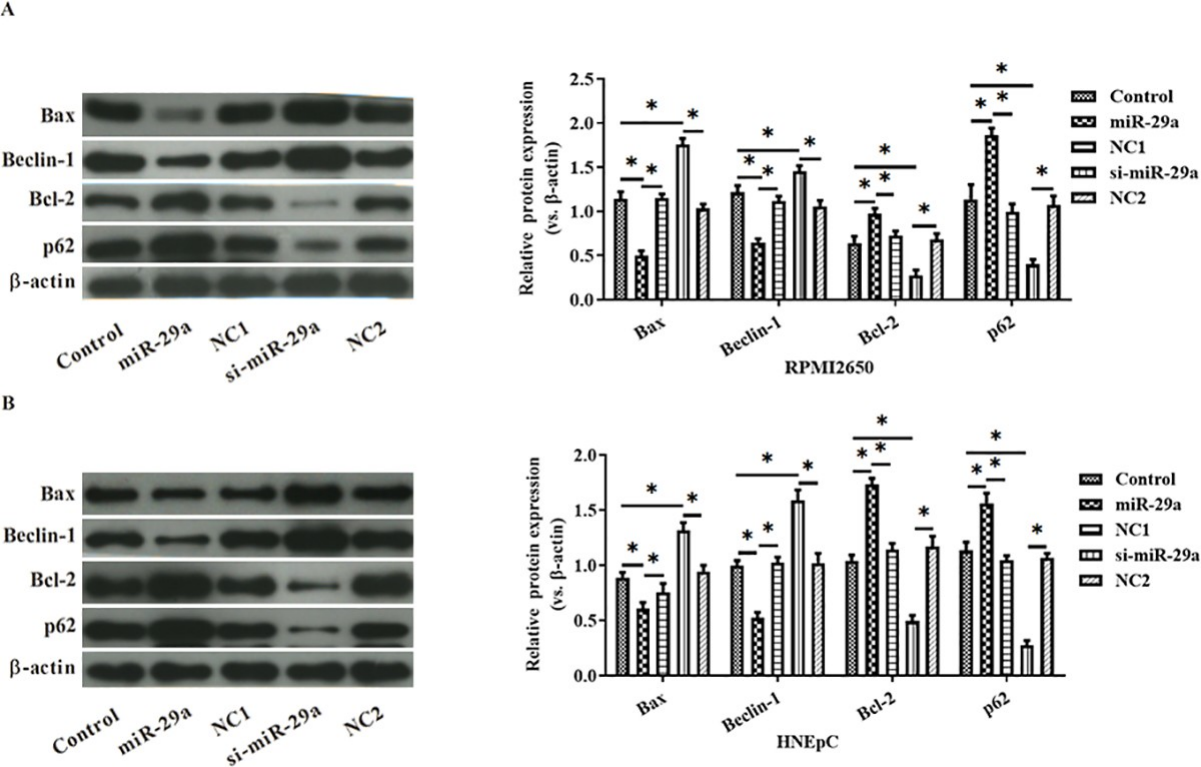
The effects of miR-29a on the expression of Bax, Beclin1, Bcl-2 and p62 in RPMI2650 cells (A) and HNEpC cells (B) were analyzed by Western Blot. *: p<0.05.

MiR-29a over-expression down-regulated the expression of Bcl-2 and Beclin1 but up-regulated that of Bax and p62 (Fig 3A and 3B).

### FOS was a target of miR-29a

**Fig 4:**
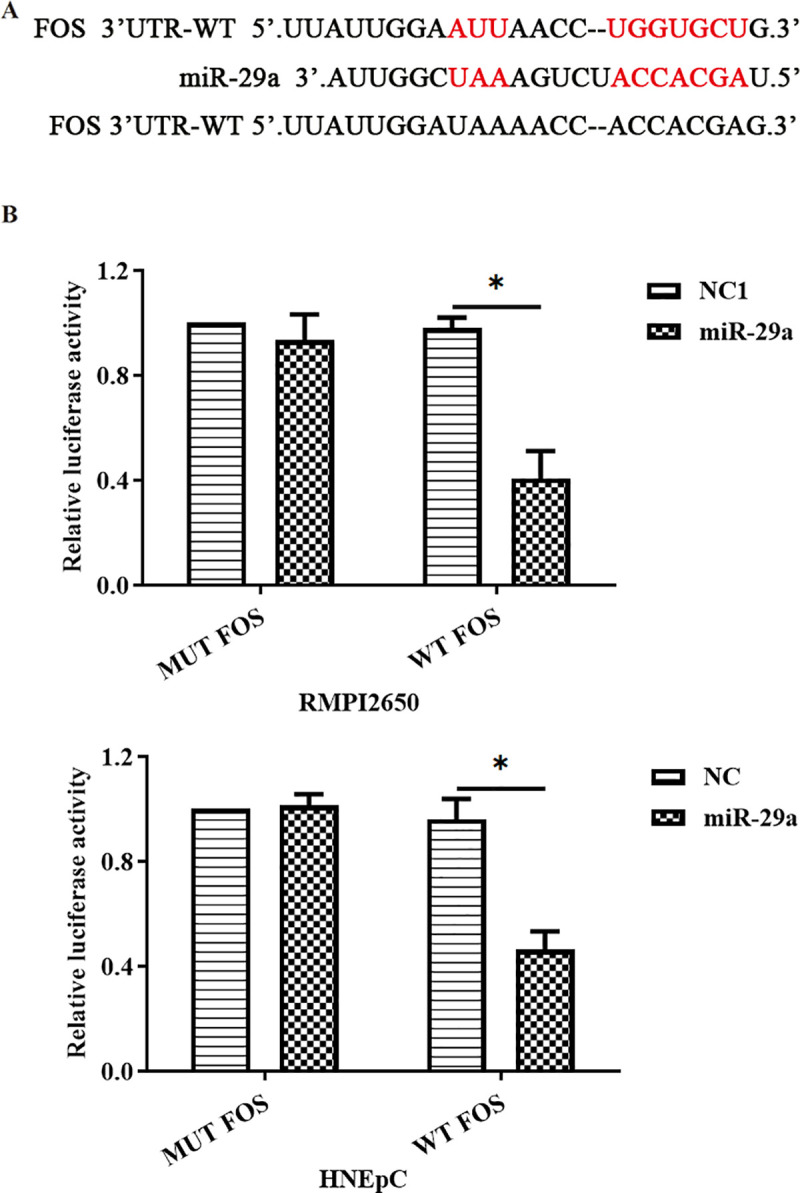
FOS was a target of miR-29a. (A) Predicted binding site of miR-29a and FOS 3’UTR; (B) results of luciferase reporter gene assay for the recombinant vector of miR-29a and FOS. *p<0.05.

To further validate whether miR-29a targeted at FOS, the luciferase reporter gene assay was performed. The results revealed that miR-29a reduced luciferase activity in FOS containing WT 3’UTR, but not in FOS containing Mut 3’UTR (Fig 4A and 4B).

MiR-29a over-expression and FOS silencing both could significantly promote cell proliferation and inhibit cell apoptosis. After transfection with miR-29a and FOS, there was a decrease in cell proliferation but an increase in cell apoptosis.

**Fig 5:**
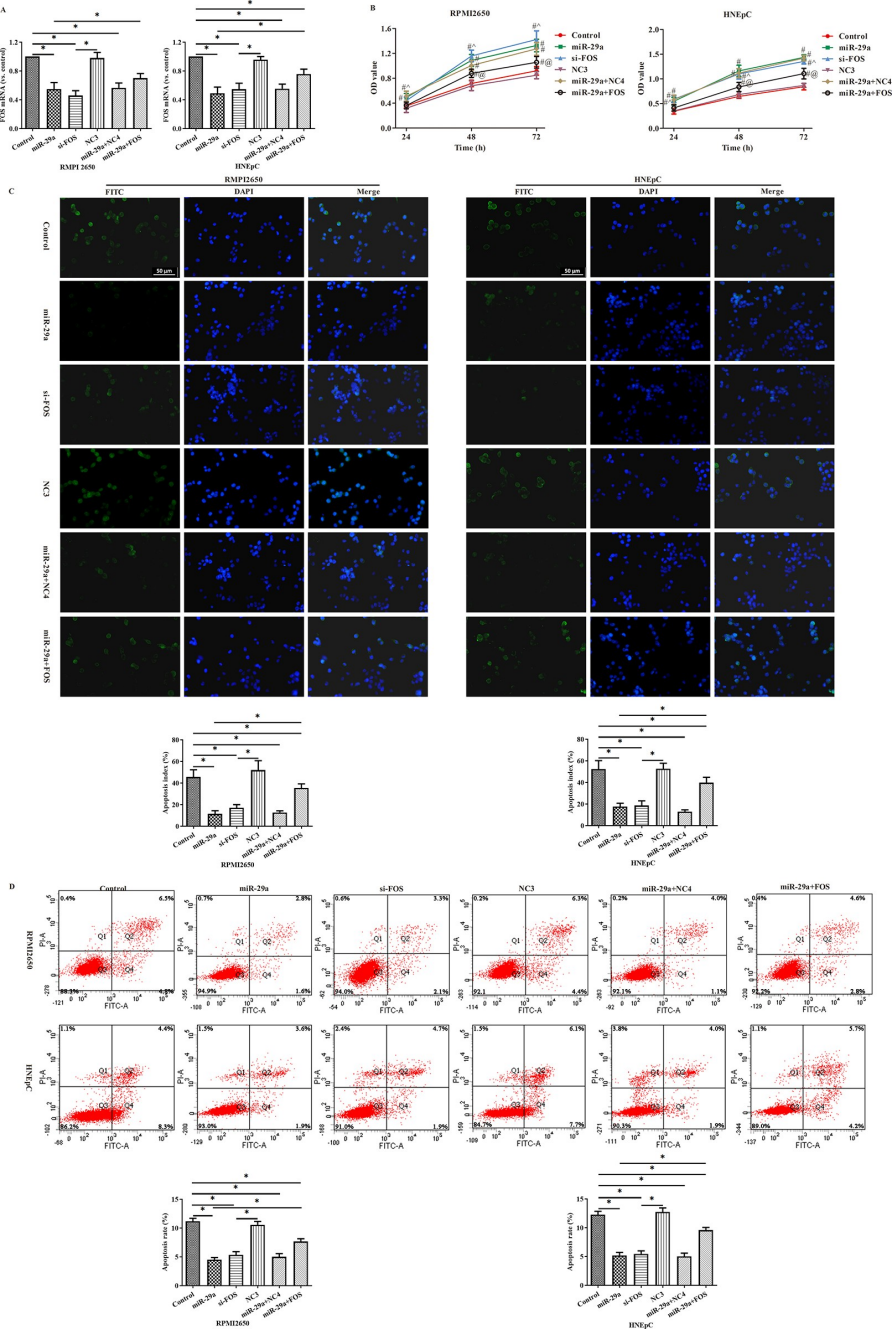
Mir-29a over-expression promoted the proliferation of RPMI2650 cells and HNEpC cells but inhibited their apoptosis by targeting at FOS. (A) FOS transfection efficiency was validated by PCR; (B) CCK8 assay; (C-D) flow cytometry and TUNEL assay. Compared with control group, #p<0.05; compared with NC3 group, ^p<0.05; compared with miR-29a group, @p<0.05. *:p<0.05.

The results showed that FOS was successfully transfected into RPMI2650 cells and HNEpC cells (Fig 5A), miR-29a over-expression and FOS silencing both could significantly promote the cell proliferation and inhibit the cell apoptosis, and there was a decrease in the cell proliferation but an increase in the cell apoptosis after transfection with miR-29a and FOS (Fig 5B–5D).

**Fig 6:**
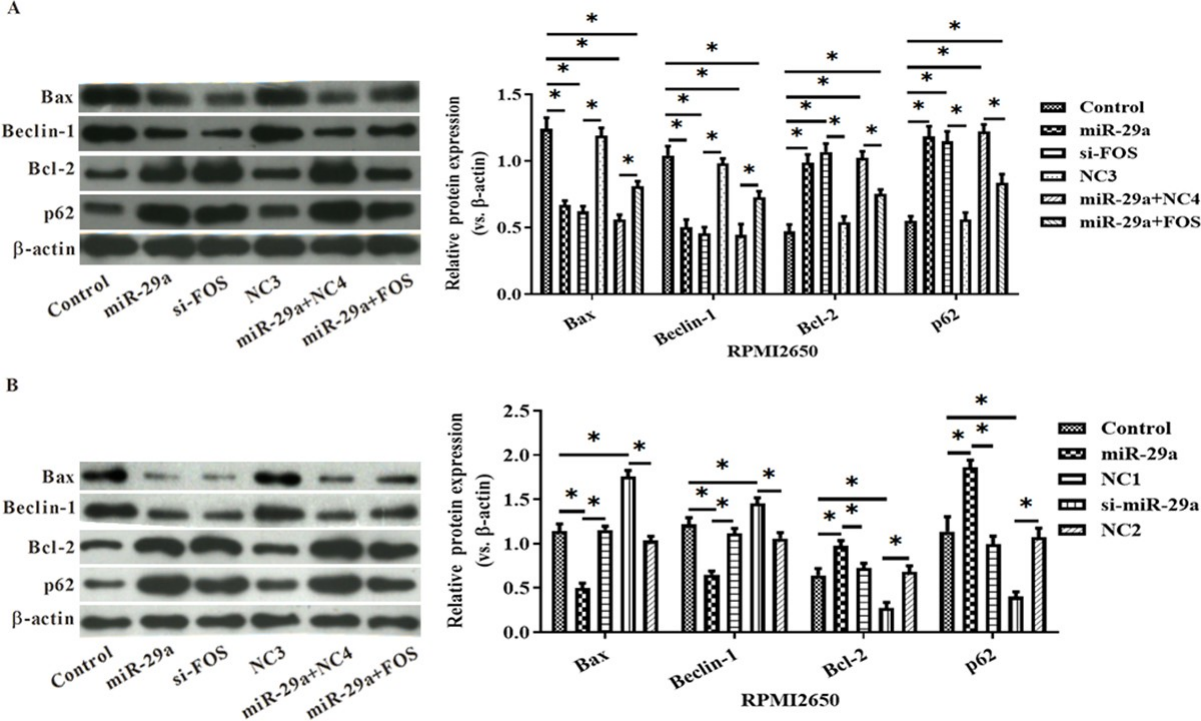
The effects of miR-29a on the expression of Bax, Beclin1, Bcl-2 and p62 in RPMI2650 cells (A) and HNEpC cells (B) were analyzed by Western Blot. *:p<0.05.

MiR-29a down-regulated the expression of Bcl-2 and Beclin1 but up-regulated that of Bax and p62 by targeting at FOS (Fig 6A and 6B).

These results give us important reminding that in the patient with allergic rhinitis both, miR-29a and FOS may be involved in regulating RPMI2650 cells and HNEpC cells of human nasal epithelial Cells proliferation and apoptosis.

## Discussion

AR is a common disease in otorhinolaryngology, and its morbidity is increasing with the development of modern industrialization; about 30% of the global population is troubled by AR [12]. Thus, it becomes highly urgent to explore new effective therapies for AR. A study showed that miRNA played a critical role in the cell development of immune system and the regulation of immune response [13].

MiR-29 family is just one of these micro non-coding RNAs, and it contains three members of hsa-miR-29b, hsa-miR-29a and hsa-miR-29c. These members are encoded by two different chromosomes and have similar performances because of their similarity in the mature sequences. MiR-29 family plays a very important role in regulating the target genes during many biological processes (e.g., cell proliferation and cell apoptosis) [14].

Although miR-29 family is involved in a wide range of biological process, most studies focus on the pathological activity of various cancers and the tumor-inhibiting activity [15,16]. The members of miR-29 family can induce cell apoptosis by increasing P53 level [17]. MiR-29b expression induces the decreased protein expression of Mcl-1 (an anti-apoptotic factor) in KMCH cells [18]. MiR-29c hinders the cell proliferation and invasion and eventually induces the cell apoptosis to target at and control c-Jun expression in endometrial cells [19]. The above evidence show that miR-29 family participates in the regulation of cell apoptosis. The occurrence of AR is associated with the abnormal apoptosis of nasal epithelial cells; the reduction in the apoptosis of epithelial cells causes the hypertrophy of nasal mucosa and thus induces such clinically typical symptoms as nasal obstruction and gland hypersecretion [20].

In this study, we confirmed that miR-29a was highly expressed in the nasal mucosa of AR patients and inhibited the apoptosis of nasal epithelial cells. In the *in vitro* experiment, miR-29a over-expression also inhibited the apoptosis of RPMI2650 cells and HNEpC cells but promoted their proliferation. Furthermore, we also proved the anti-apoptotic role of miR-29a by investigating the down-regulation and up-regulation of Bcl-2 and Beclin separately on the expression of Bax and p62.

FOS (a FBJ murine osteosarcoma viral oncogene homolog), also known as c-FOS, can interact with JUN (a primary proto-oncogene, c-JUN) to form activator protein 1 (AP-1, a transcriptional factor), i.e., it is critical for cells to adapt to the environmental changes [21] and involved in many processes including cell proliferation, differentiation and apoptosis [22]. The expression of FOS (a differentially expressed gene screened out preliminarily by us) was down-regulated in AR, which is consistent with the findings of Liu Y et al that FOS can be used as a candidate target to treat seasonable AR (SAR) in the pollen season [23].

Some studies suggested that miR-129 could suppress c-FOS expression by inhibiting MAPK signaling pathway and thus hinder the occurrence and development of epilepsy [24]. Our TargetScan prediction revealed that FOS was a potential target of miR-29a. We found that FOS protein expression level was significantly lower in the nasal tissues from AR patients than in those from normal controls (p<0.05). Interestingly, there was a negative correlation in the expression between FOS and miR-29a (r = -0.8102). Our subsequent luciferase reporter gene assay also proved that the target of miR-29a was FOS. In RPMI2650 cells and HNEpC cells, miR-29a over-expression and FOS silencing both could evidently promote cell proliferation and inhibit cell apoptosis. After transfection with miR-29a and FOS, however, we observed a decrease in cell proliferation but an increase in cell apoptosis. This further indicates that miR-29a/FOS axis is present in the development process of AR, and miR-29a can inhibit the apoptosis of nasal cells and promote their proliferation via FOS pathway.

Nasal mucosal epithelial cells are over-proliferated as a result of a local allergic reaction in patients who with the allergic rhinitis. This study verified that the role of FOS in allergic rhinitis nasal epithelial cell proliferation (apoptosis) effects at a cellular level, and verified the results in the gene expression profile of nasal mucosal tissue in previous experiments. In addition, we also researched the regulation mechanism of FOS. But we know that’s not enough. we will continue to explore the relationship among immune reaction, allergic reaction and MiR-29a/FOS in the later study.

## Conclusion

This study has some limitations. MiR-29a has many other targets, and some of them may mediate the proliferation and apoptosis of human nasal cells parallel to FOS, which will be focused on our incoming study. Our next research emphasis is the testing of a larger sample size and the establishment of an animal model for deeper investigation. However, miR-29a-/FOS axis still can be regarded as a potential marker and a new therapy for AR.

## Supporting information

S1 Raw images (PDF)
