# Correction: MicroRNA-29a promotes the proliferation of human nasal epithelial cells and inhibits their apoptosis and promotes the development of allergic rhinitis by down-regulating FOS expression

**DOI:** 10.1371/journal.pone.0302708

**Published:** 2024-04-19

**Authors:** The PLOS ONE Staff

The seventh and eighth authors are listed out of order. The publisher apologizes for the error. The correct order is: Yuqin Fan ^1^, Zhiyuan Tang ^2^, Jie Sun ^3^, Xiaorui Zhao ^4^, Zhen Li ^4^, Yiqing Zheng ^5^, Juan-Feng ^4^, Xianhai Zeng^2^

**1** Department of Otolaryngology-Head and Neck Surgery, Shanghai Ninth People’s Hospital, Shanghai Jiao Tong University School of Medicine, Ear Institute, Shanghai Jiao Tong University School of Medicine, Shanghai Key Laboratory of Translational Medicine on Ear and Nose Diseases, Shanghai, China, **2** Department of Otorhinolaryngology, Shenzhen Longgang E.N.T hospital & Shenzhen Key Laboratory of E.N.T., Institute of E.N.T., Shenzhen, Guangdong, China, **3** Department of Otorhinolaryngology, The Eighth Affiliated Hospital of Sun Yat-sen University, Shenzhen, Guangdong, China, **4** Department of Otorhinolaryngology, The First Affiliated Hospital of Xinjiang Medical University, Urumqi, Xinjiang, China, **5** Department of Otolaryngology, Sun Yat-Sen Memorial Hospital, Guangzhou, Guangdong, P.R.China
