# Supplementary material for: MicroRNA-29a promotes the proliferation of human nasal epithelial cells and inhibits their apoptosis and promotes the development of allergic rhinitis by down-regulating FOS expression
Source: PLoS One. 2021 Aug 12;16(8):e0255480. doi: 10.1371/journal.pone.0255480 (PMC8360612; doi:10.1371/journal.pone.0255480)
Supplement: S1 Raw images — (PDF) [file pone.0255480.s001.pdf]

Fig.1D:FOS protein expression in the nasal tissues from AR patients and healthy controls was analyzed by western Blot.

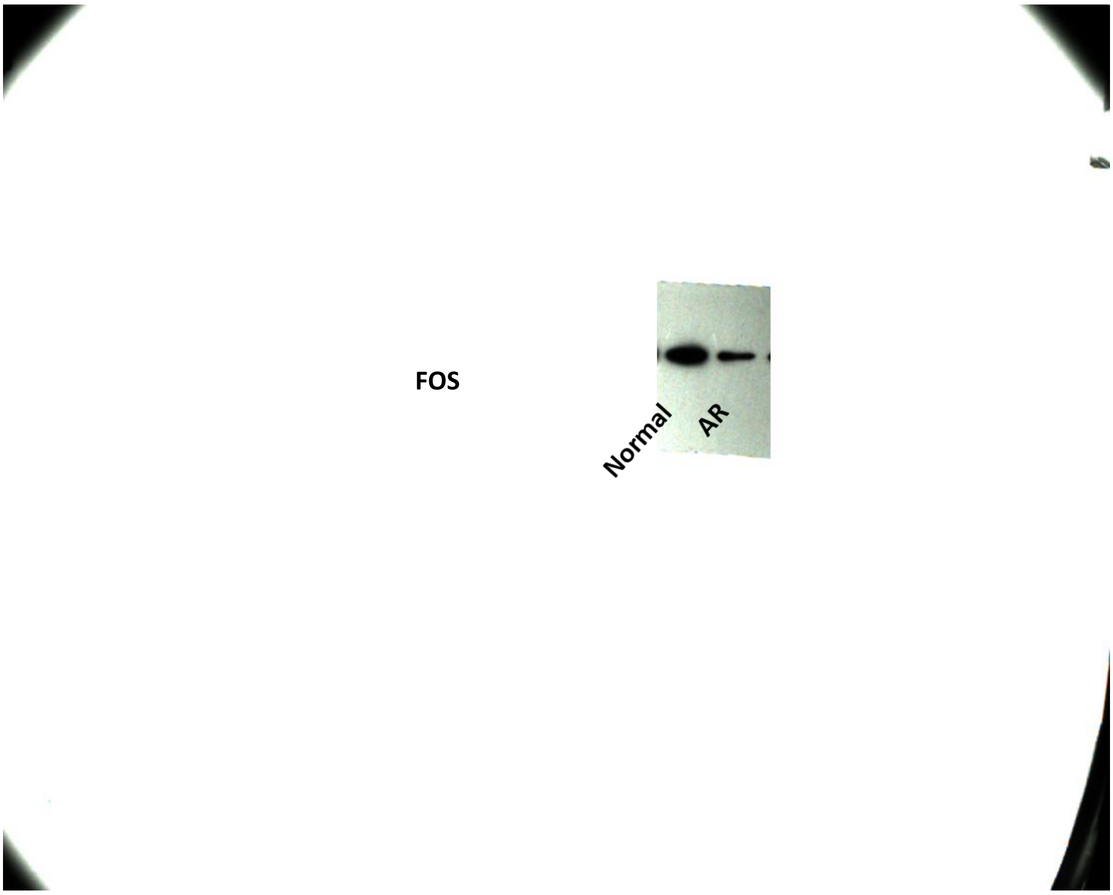

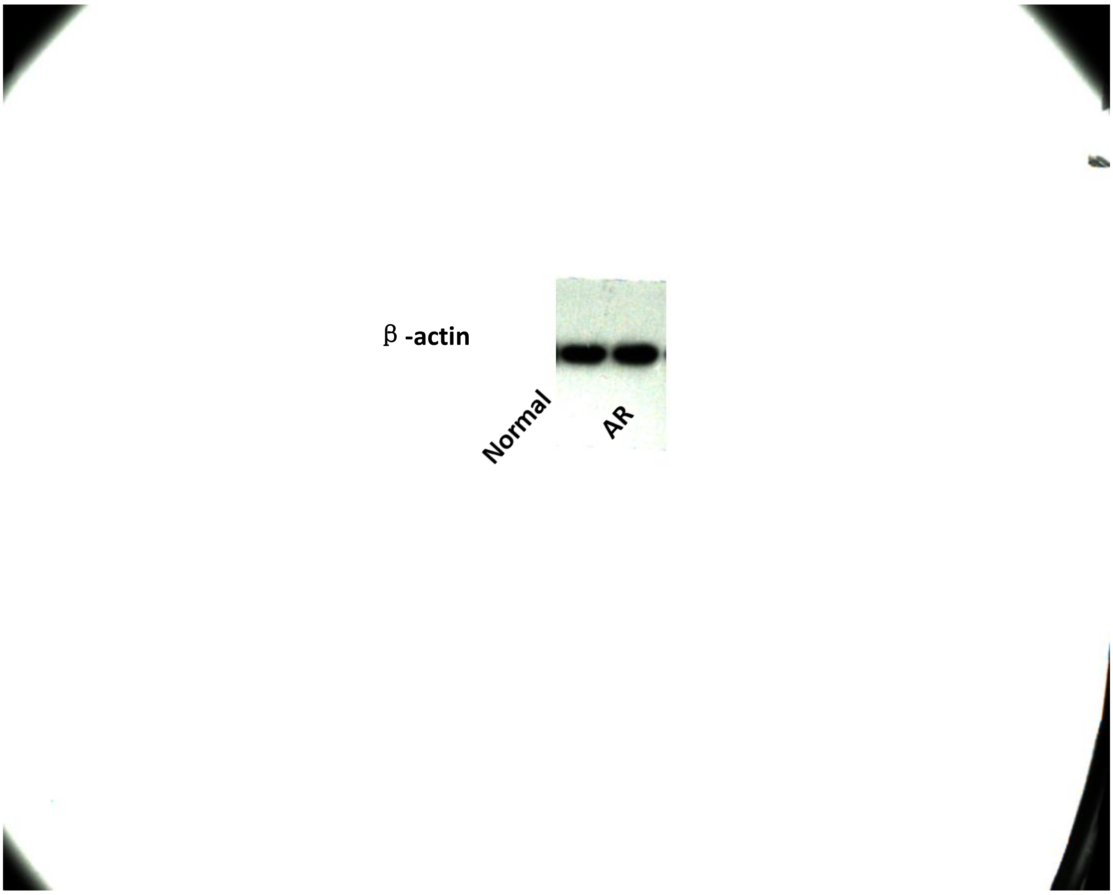

Fig.3A:The effects of miR-29a on the expression of Bax, Beclin1, Bcl-2 and p62 in RPMI2650 cells were analyzed by Western Blot.

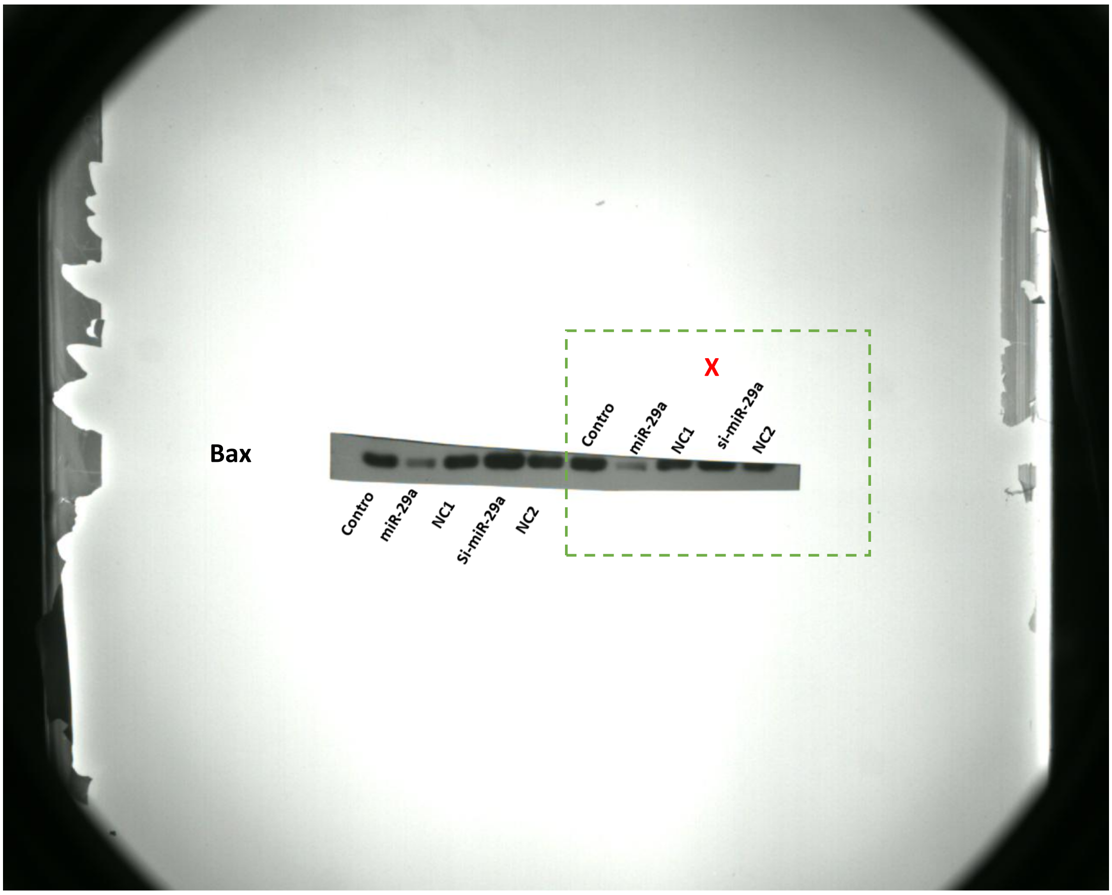
Bax
Contro
miR-29a
NC1
Si-miR-29a
NC2
X
Contro
miR-29a
NC1
si-miR-29a
NC2

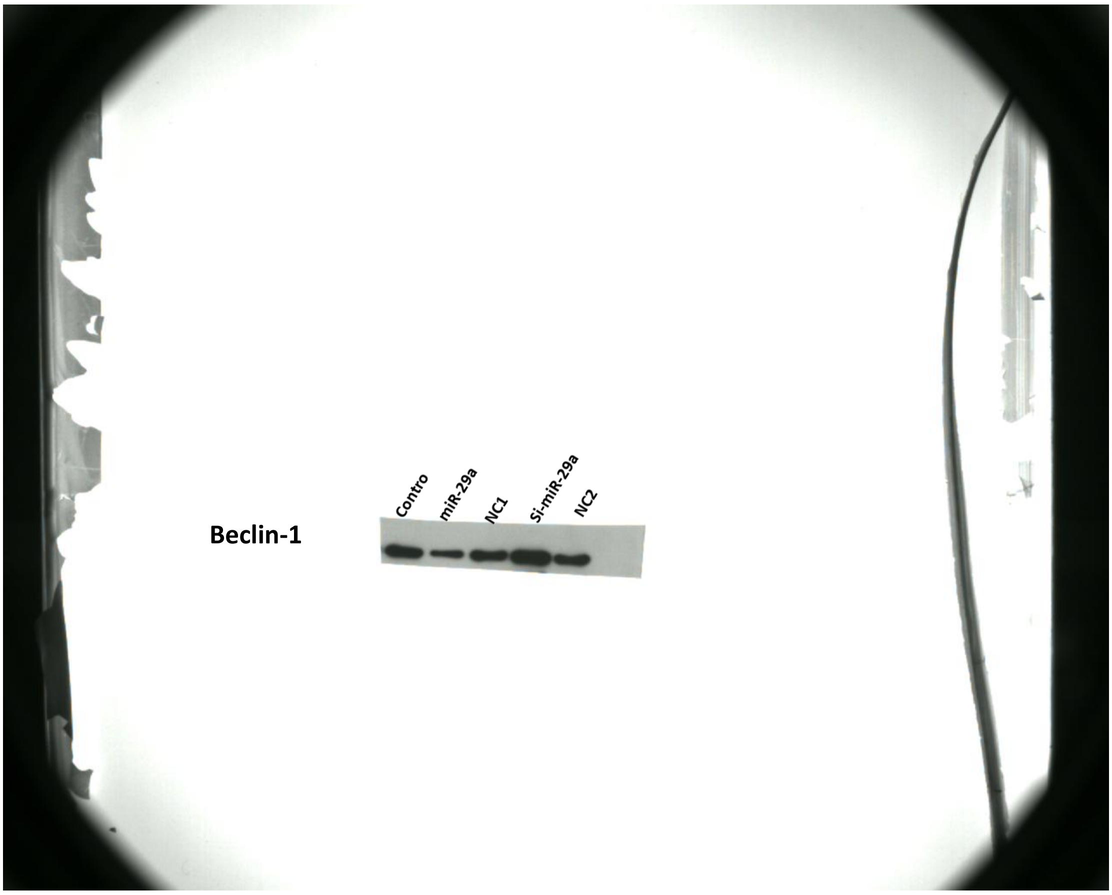
Beclin-1
Contro
miR-29a
NC1
Si-miR-29a
NC2

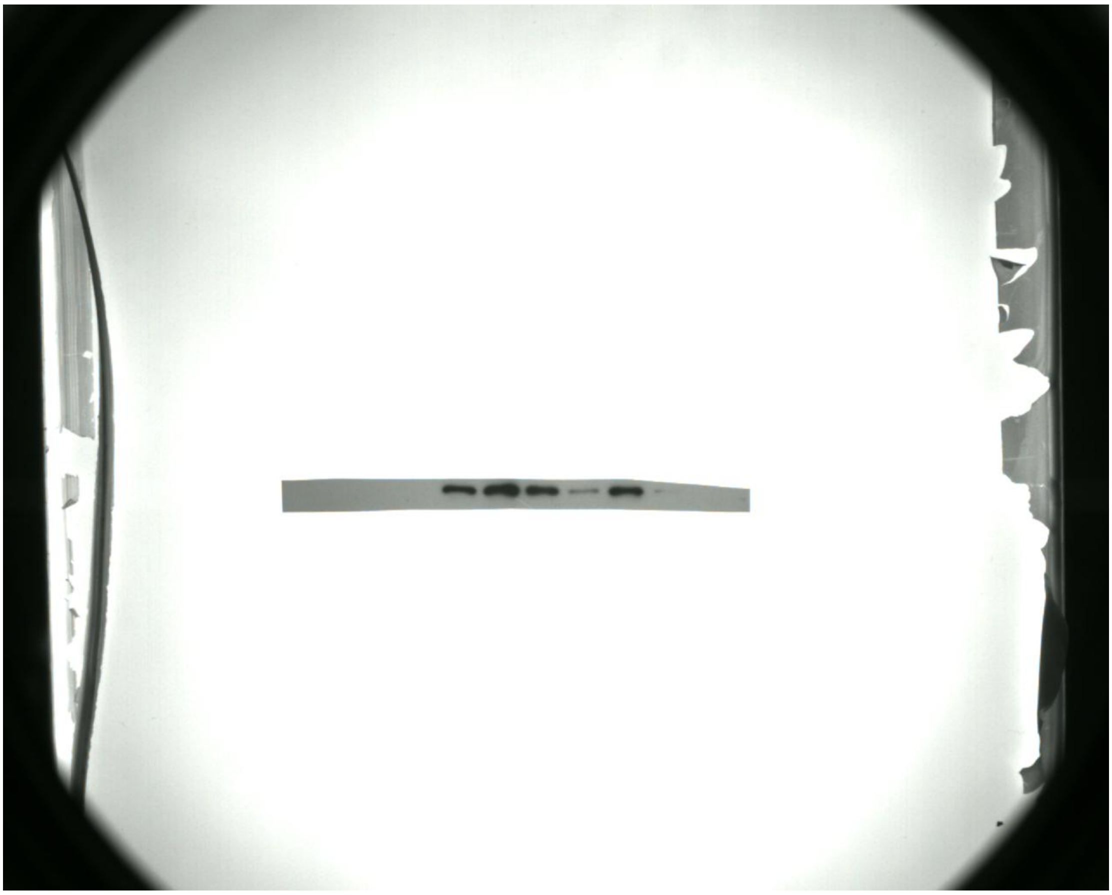

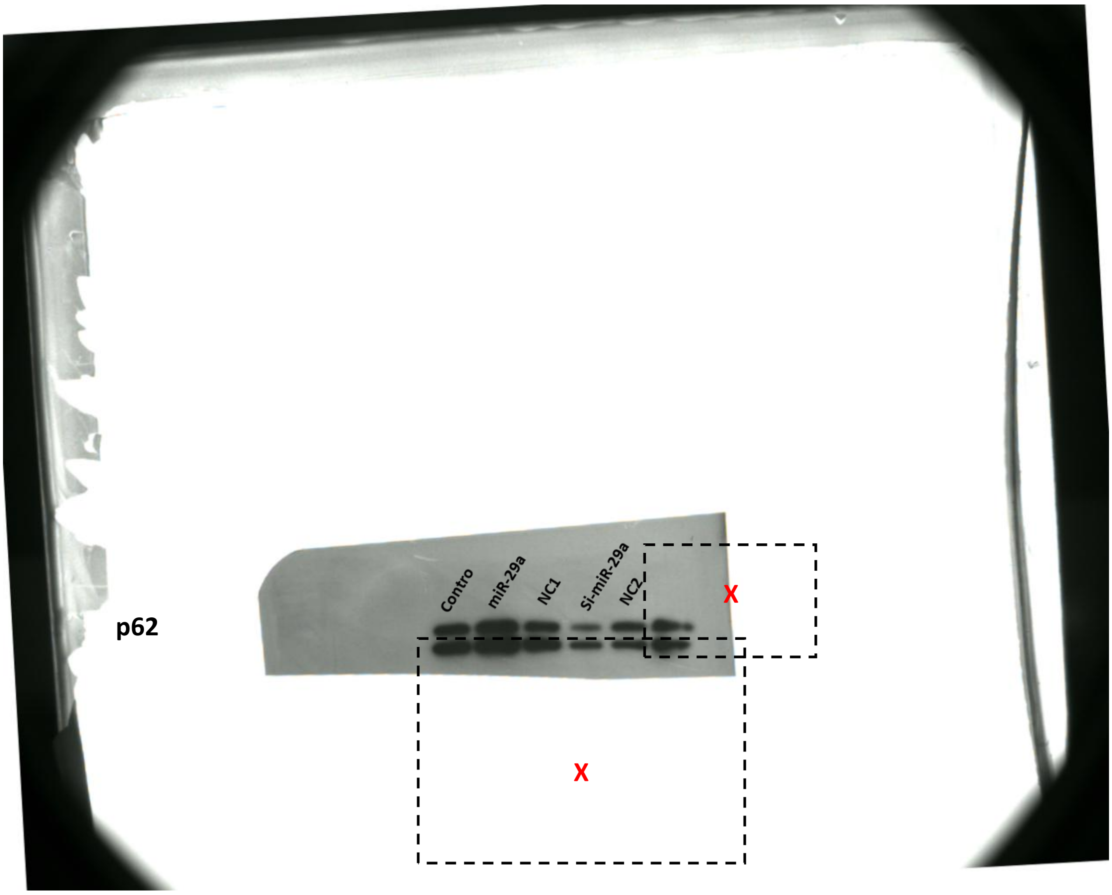
p62
Contro
miR-29a
NC1
Si-miR-29a
NC2
X
X

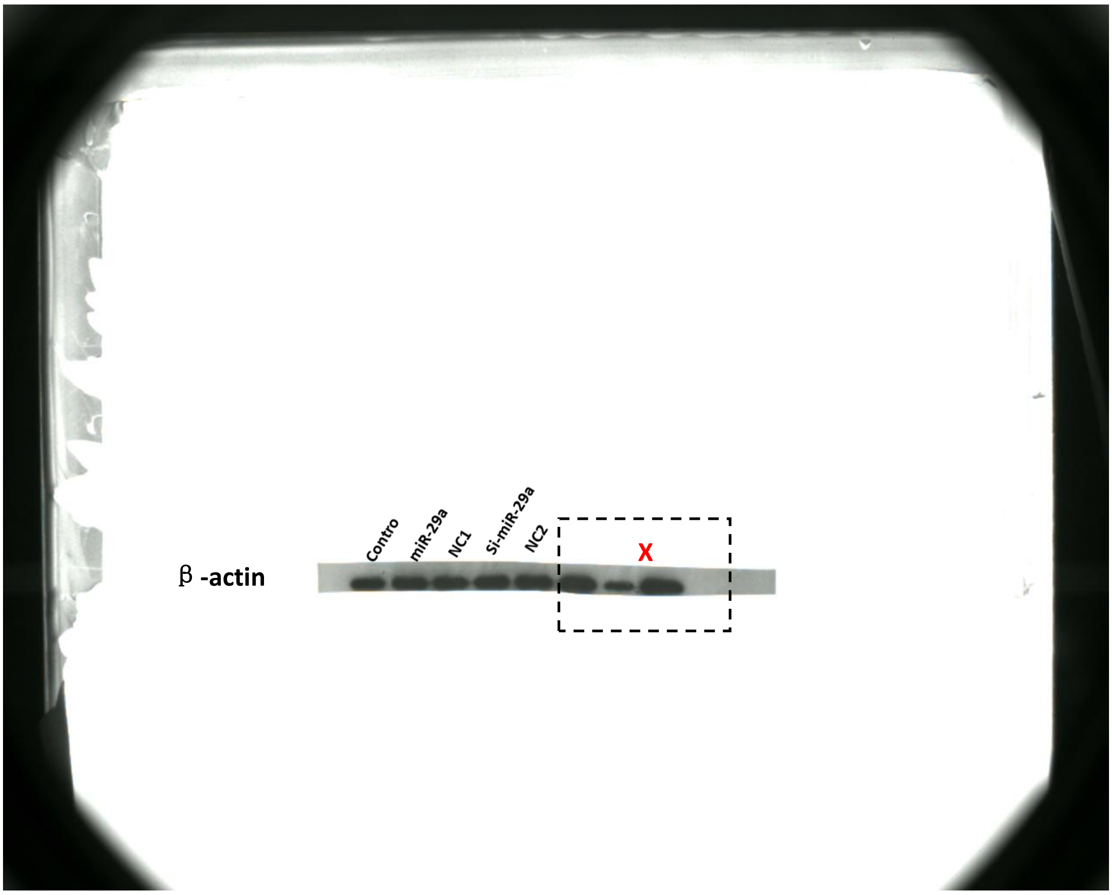

Fig.3B:The effects of miR-29a on the expression of Bax, Beclin1, Bcl-2 and p62 in HNEpC cells were analyzed by Western Blot.

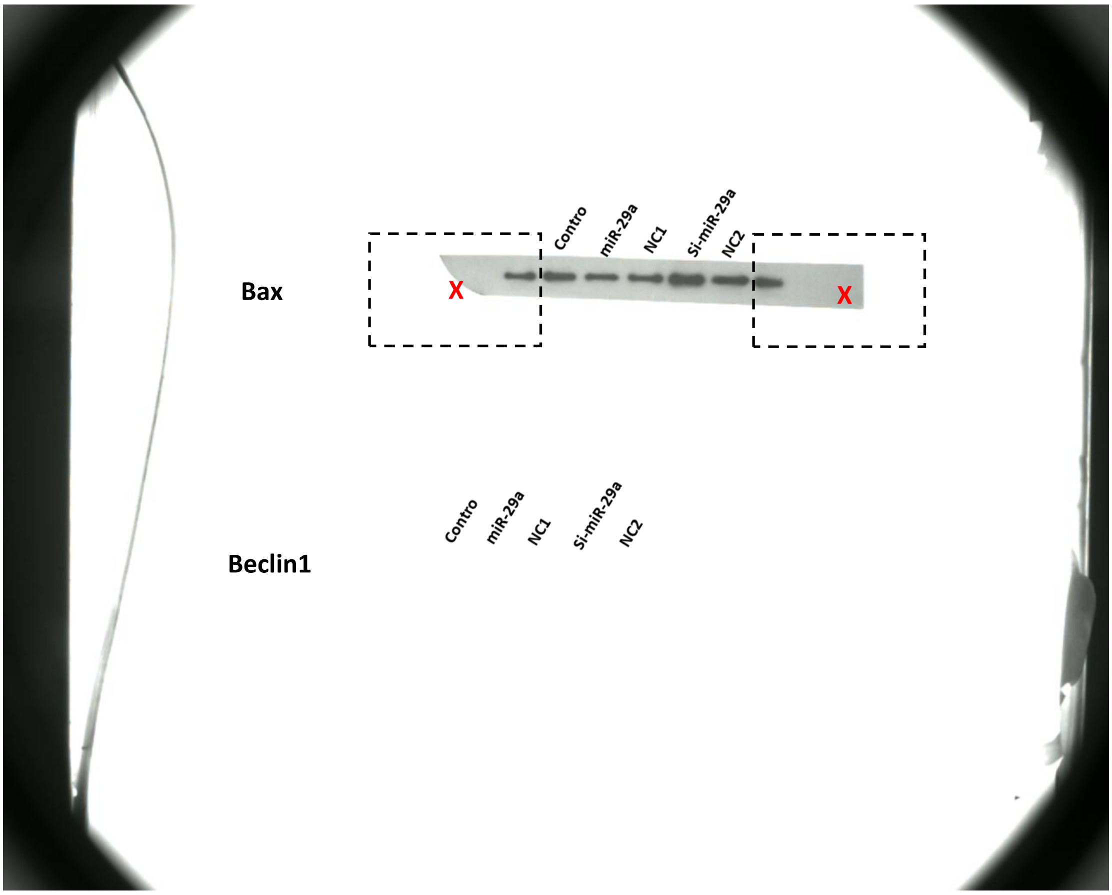
Bax
Contro
miR-29a
NC1
Si-miR-29a
NC2
X
X
Beclin1
Contro
miR-29a
NC1
Si-miR-29a
NC2

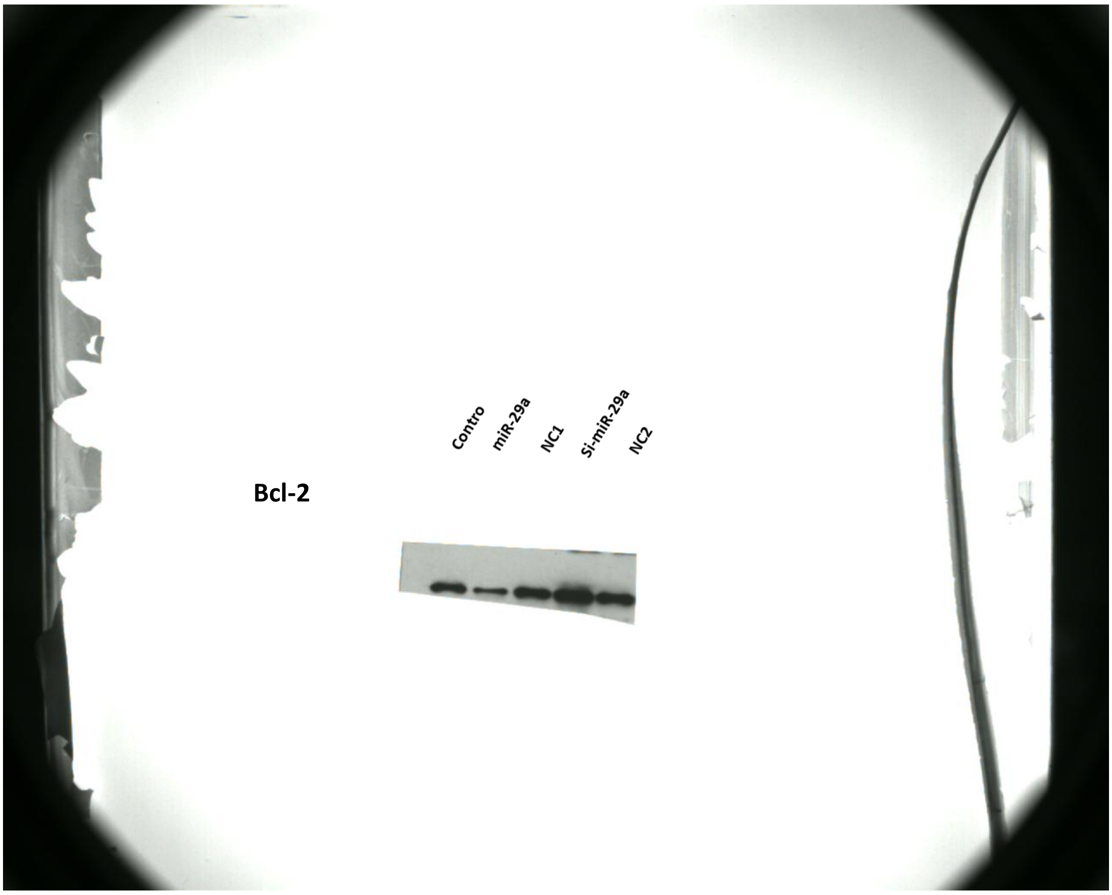
Bcl-2
Contro
miR-29a
NC1
Si-miR-29a
NC2

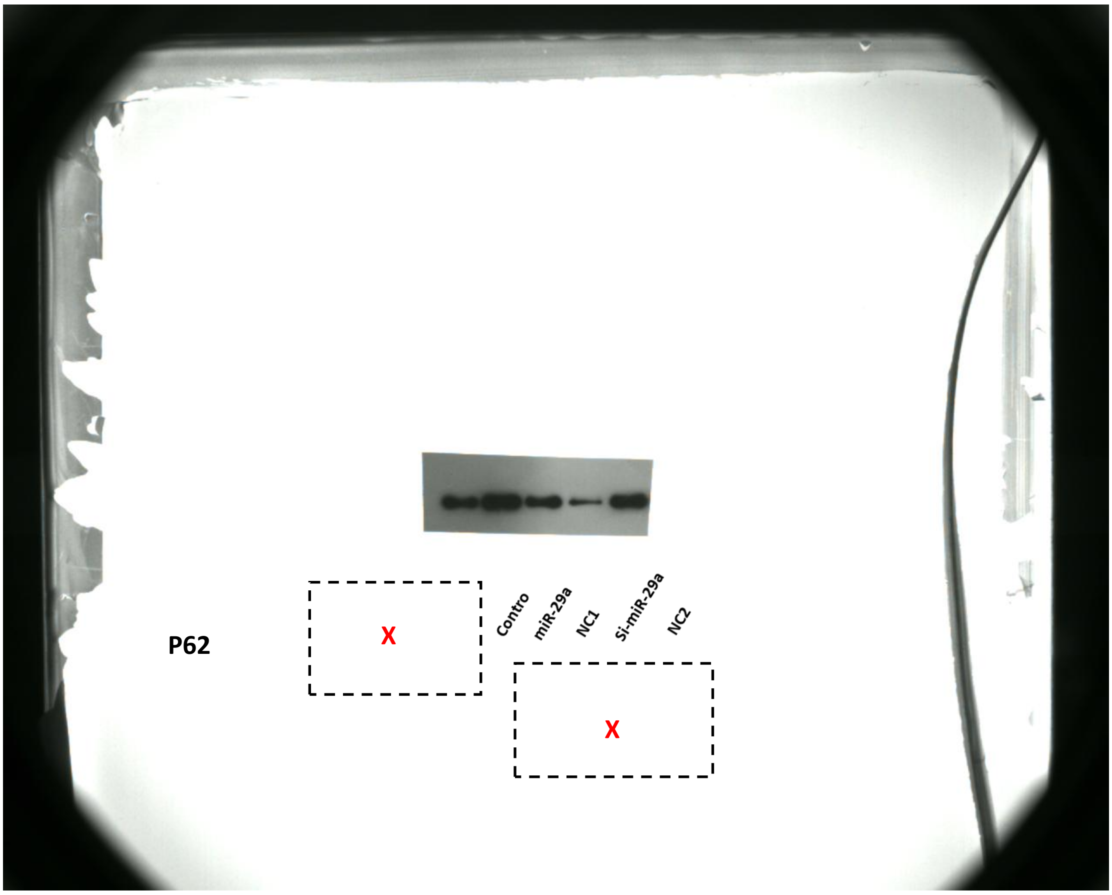
P62
X
Contro
miR-29a
NC1
Si-miR-29a
NC2
X

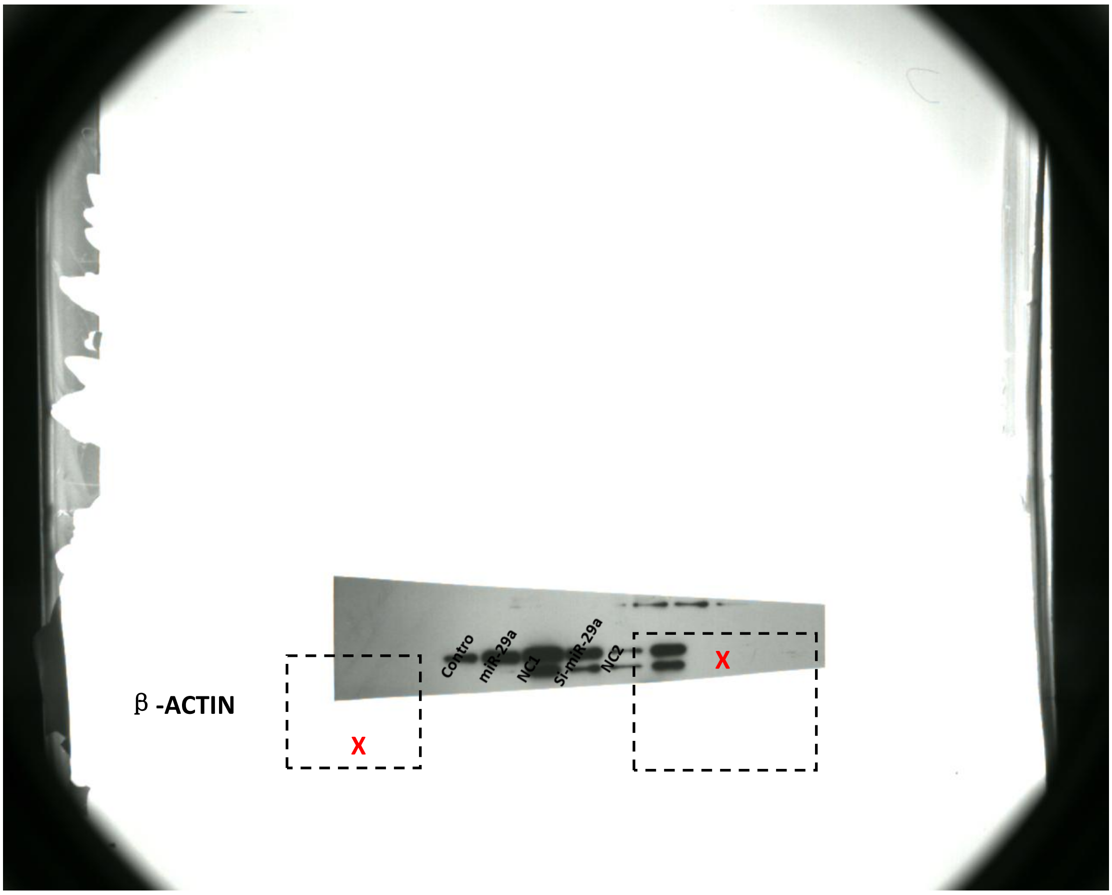
β -ACTIN
X
Contro
miR-29a
NC1
Si-miR-29a
NC2
X

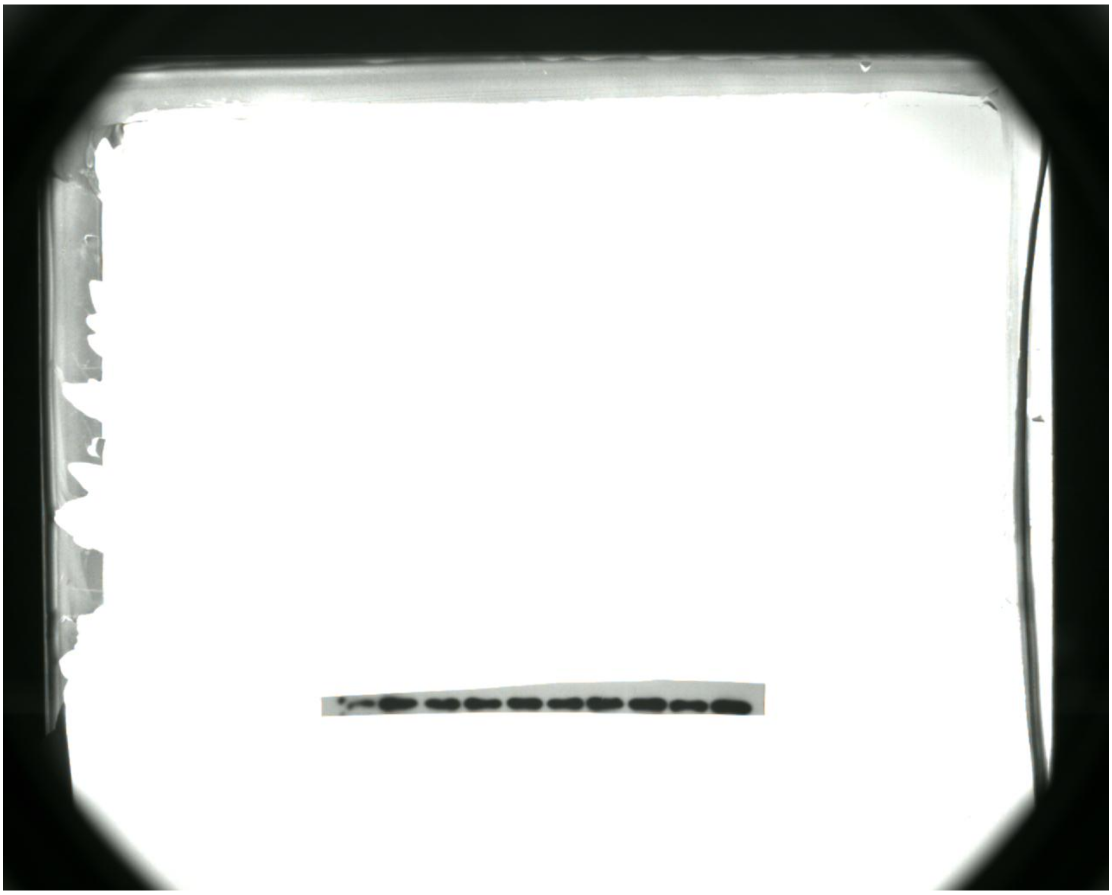

Fig. 6A. The effects of miR-29a on the expression of Bax, Beclin1, Bcl-2 and p62 in RPMI2650 cells were analyzed by Western Blot.

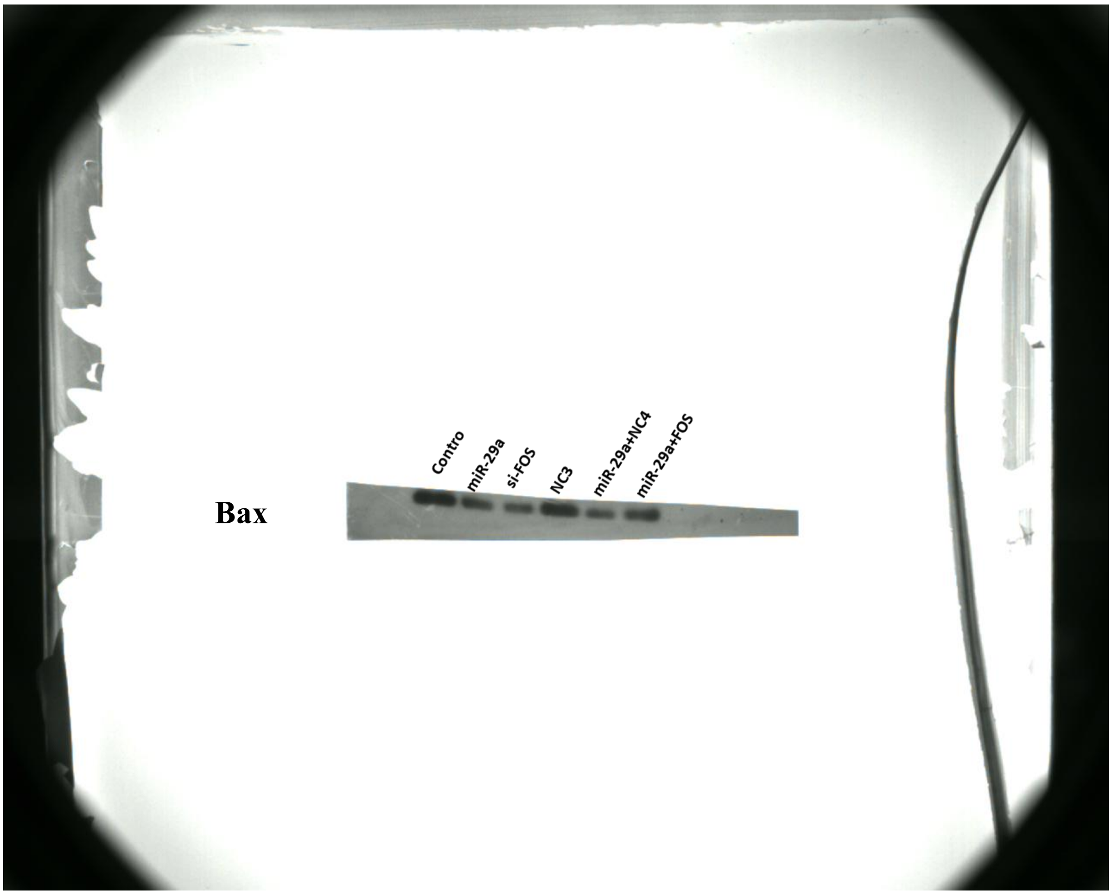
Contro
miR-29a
si-FOS
NC3
miR-29a+NC4
miR-29a+FOS
Bax

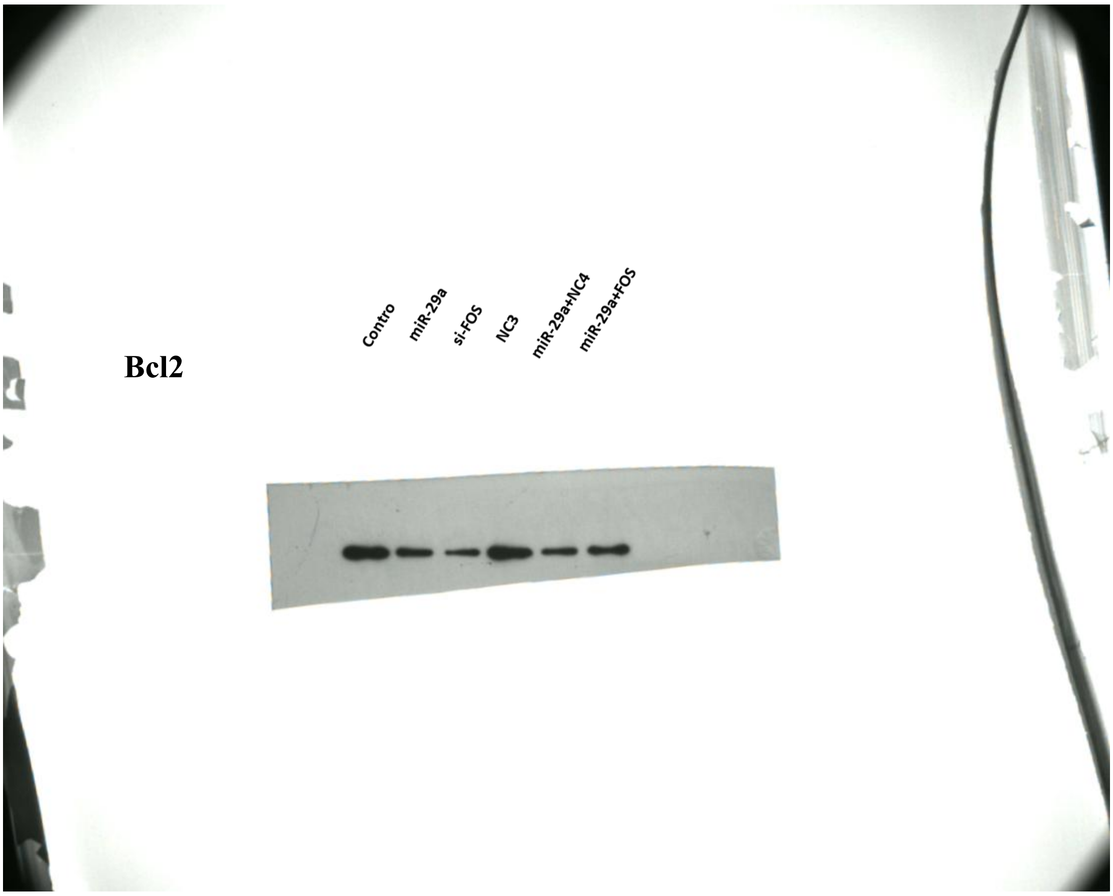
Contro
miR-29a
si-FOS
NC3
miR-29a+NC4
miR-29a+FOS
Bcl2

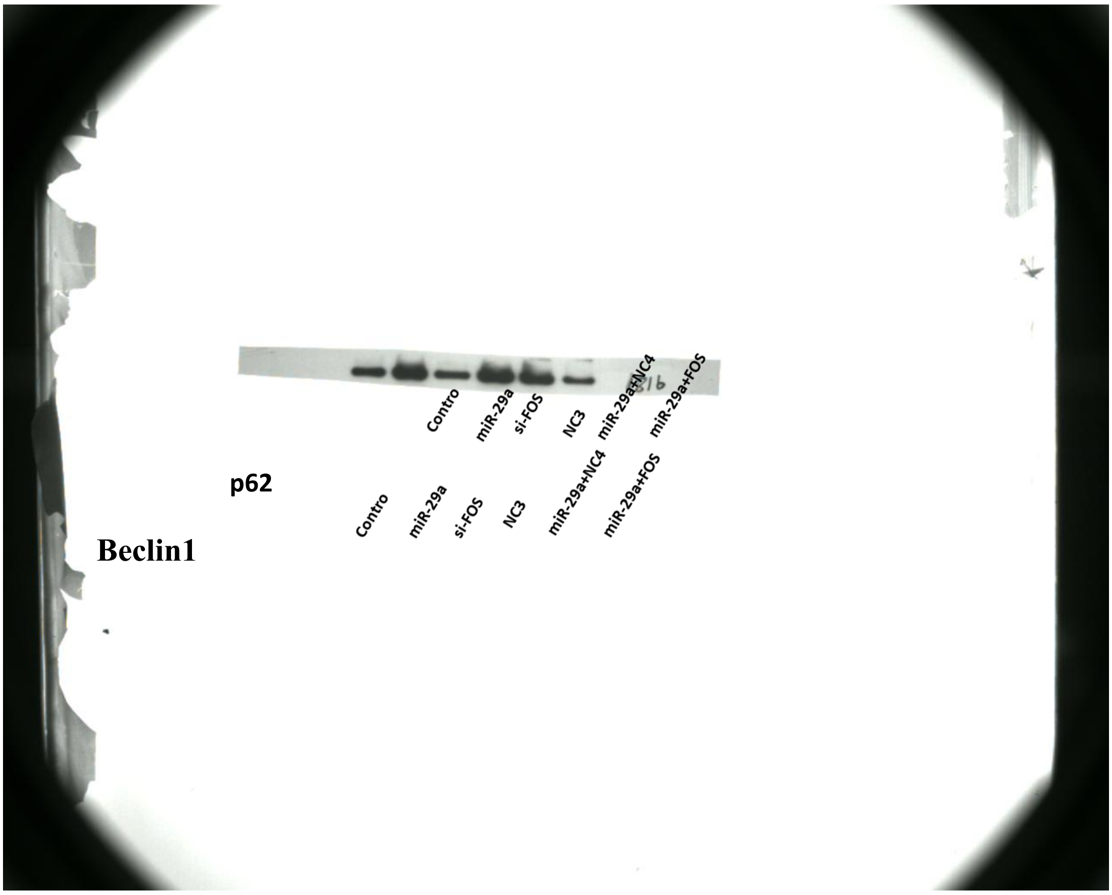
Contro
miR-29a
si-FOS
NC3
miR-29a+NC4
miR-29a+FOS
p62
Contro
miR-29a
si-FOS
NC3
miR-29a+NC4
miR-29a+FOS
Beclin1

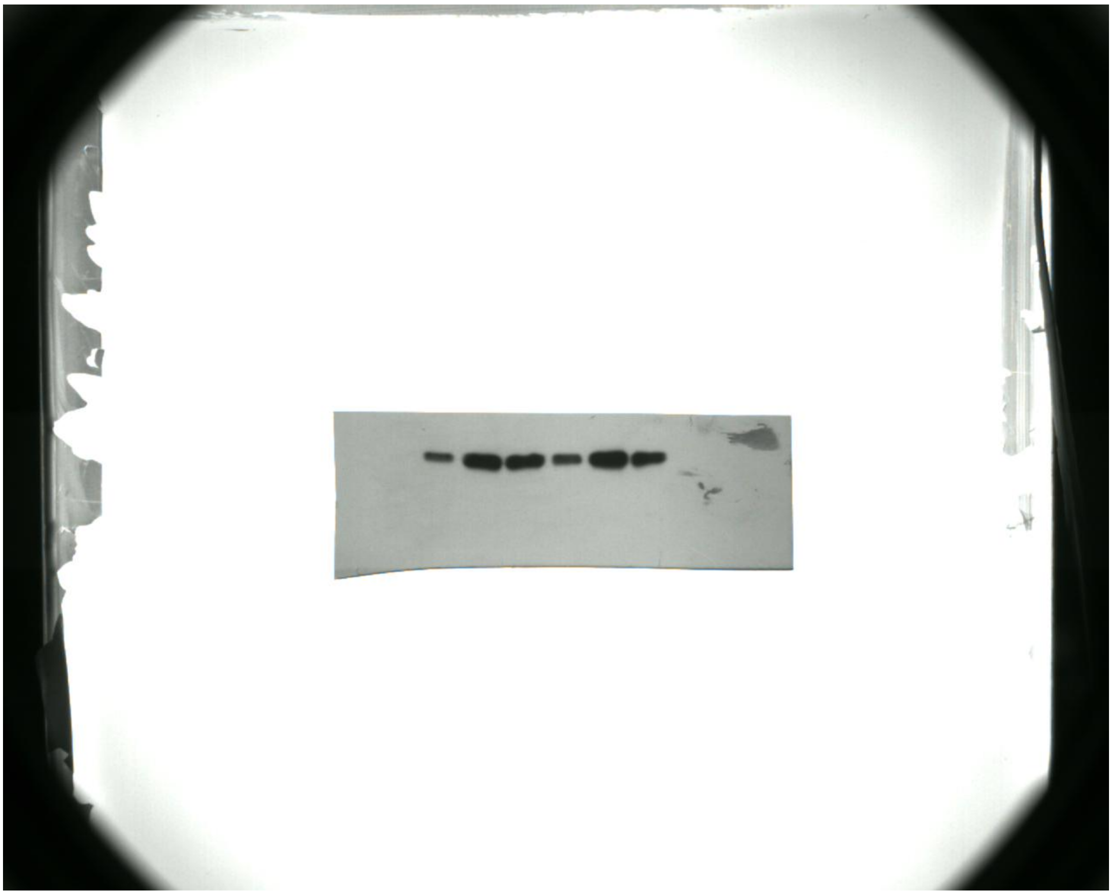

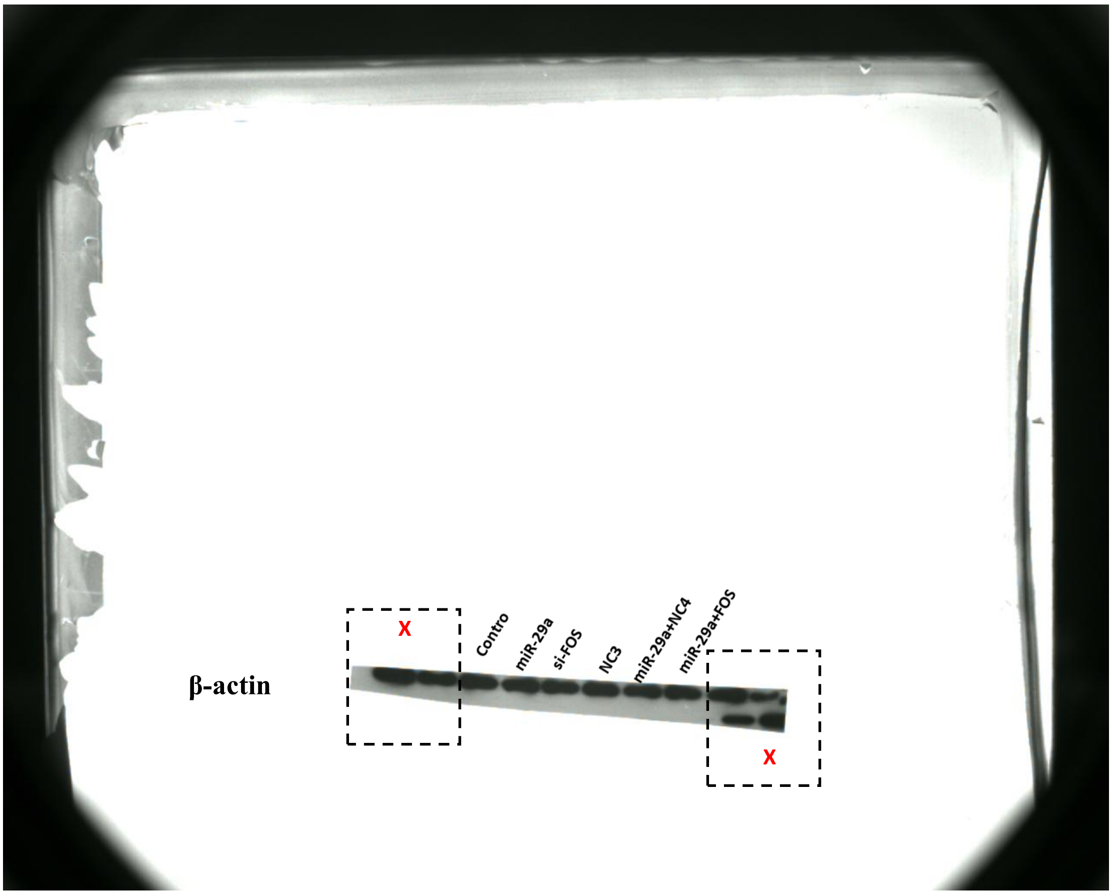

Fig. 6B. The effects of miR-29a on the expression of Bax, Beclin1, Bcl-2 and p62 in HNEpC cells were analyzed by Western Blot.

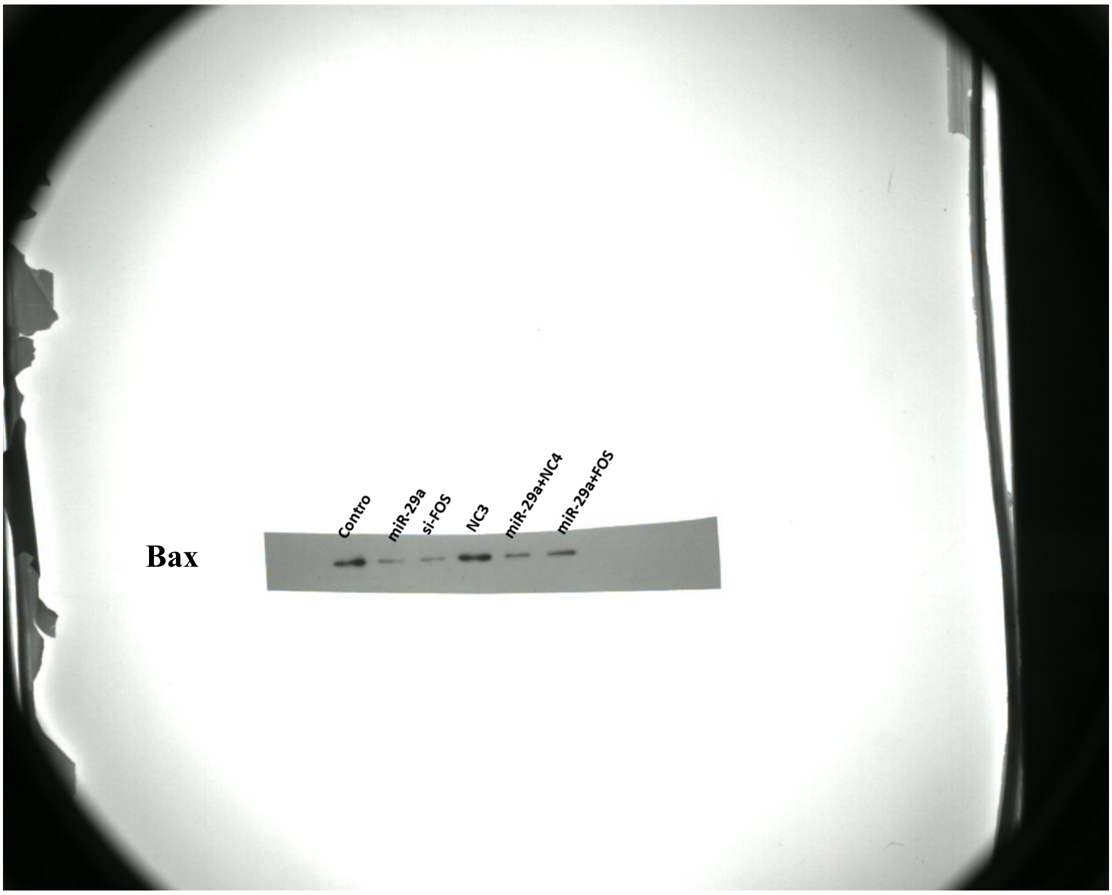
Bax
Contro
miR-29a
si-FOS
NC3
miR-29a+NC4
miR-29a+FOS

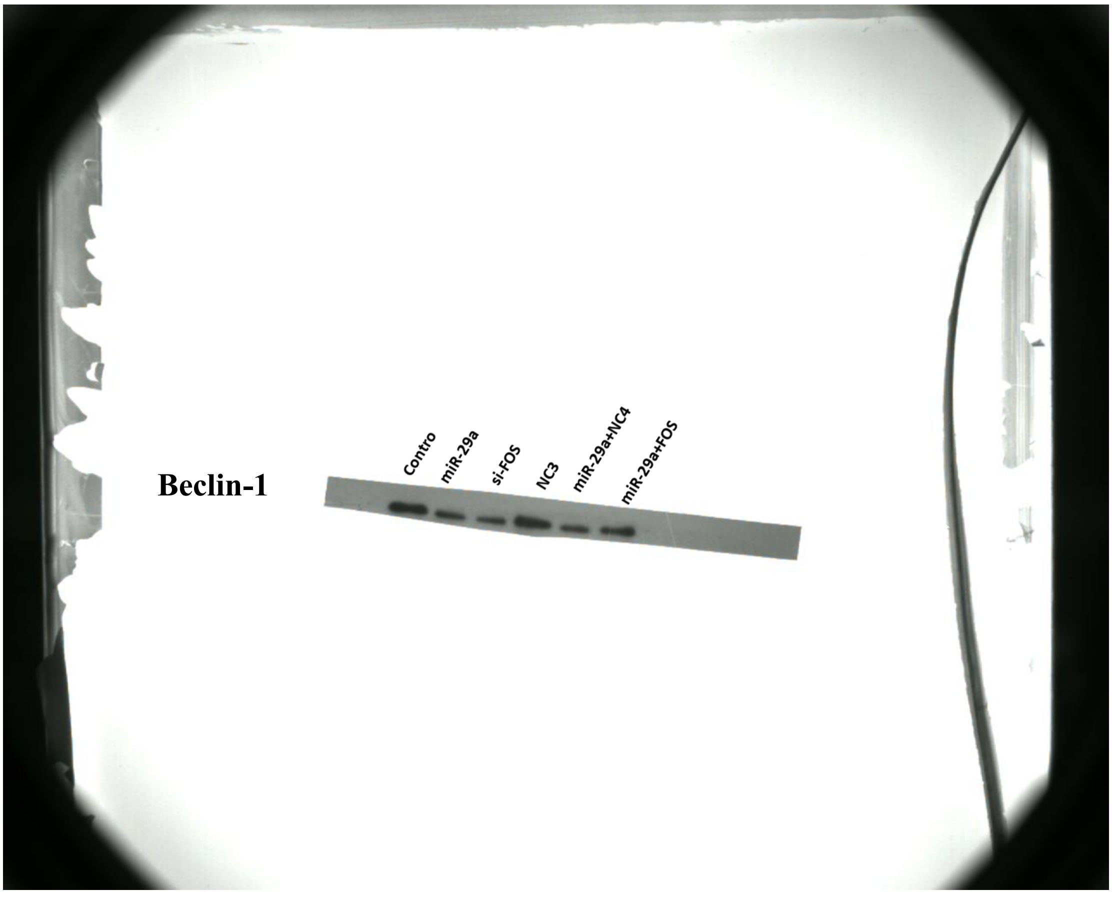
Beclin-1
Contro
miR-29a
si-FOS
NC3
miR-29a+NC4
miR-29a+FOS

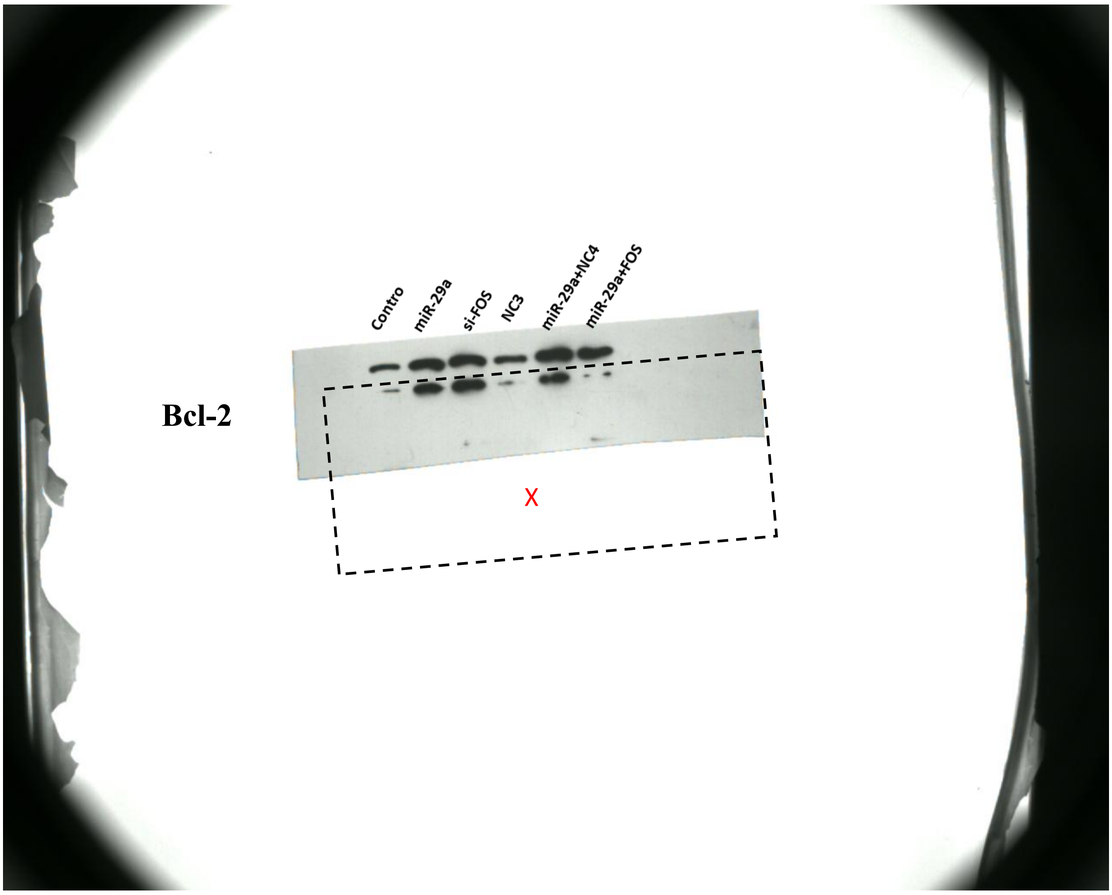
Contro
miR-29a
si-FOS
NC3
miR-29a+NC4
miR-29a+FOS
Bcl-2
X

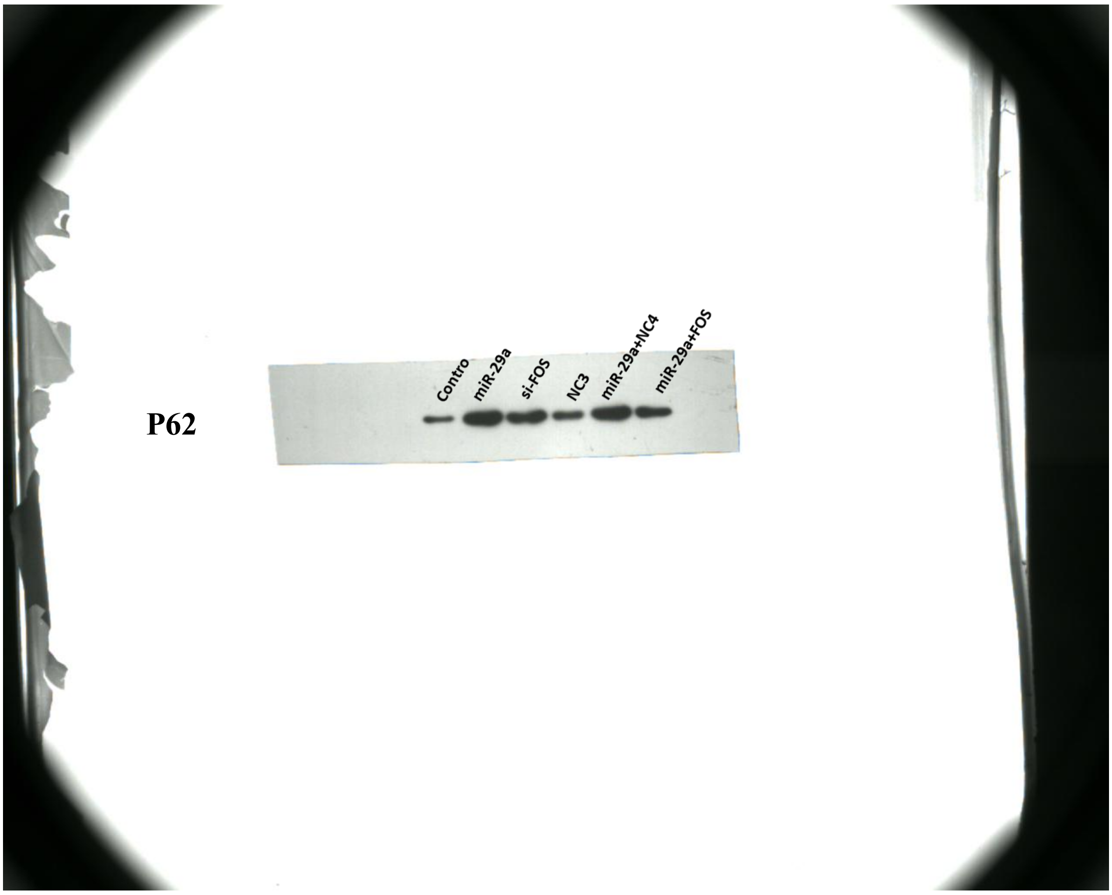
Contro
miR-29a
si-FOS
NC3
miR-29a+NC4
miR-29a+FOS
P62

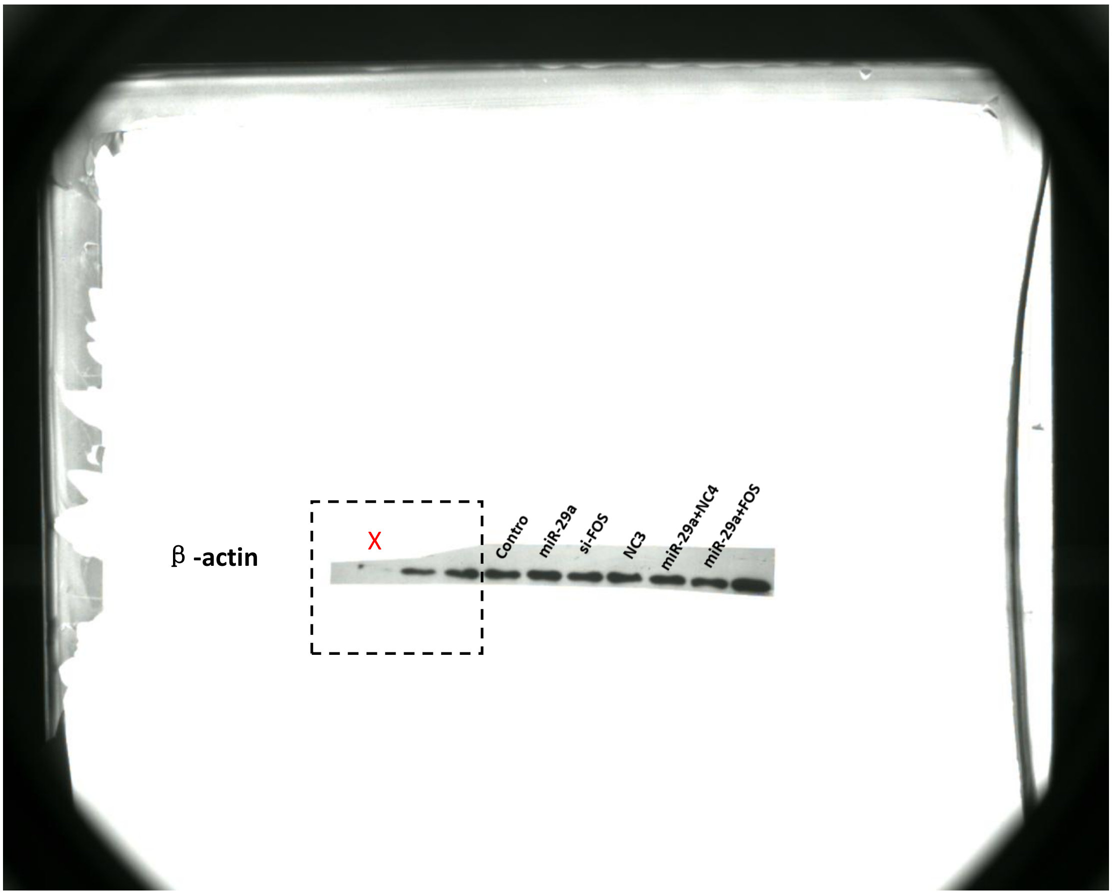
β -actin
X
Contro
miR-29a
si-FOS
NC3
miR-29a+NC4
miR-29a+FOS
